# Performance of a remote interrogation system for the in-hospital evaluation of cardiac implantable electronic devices

**DOI:** 10.1007/s10840-015-0091-4

**Published:** 2015-12-22

**Authors:** Suneet Mittal, Kevin Younge, Kelly King-Ellison, Eric Hammill, Kenneth Stein

**Affiliations:** The Arrhythmia Institute at the Valley Hospital, 223 North Van Dien Avenue, Ridgewood, NJ 07450 USA; Boston Scientific, Minneapolis, MN USA

**Keywords:** Cardiac implantable electronic device, Interrogation, Performance, Remote evaluation

## Abstract

**Purpose:**

Patients with a cardiac implantable electronic device (CIED) often need device interrogation in an in-hospital environment. A diagnosis-only, remote interrogation device and process for CIED interrogation was developed to address this situation. Here, we describe our initial clinical experience with this system.

**Methods:**

The LATITUDE Consult Communicator is a stand-alone *interrogation-only* device used to read the patient’s implanted CIED. Once retrieved, the data are securely transmitted via an analog phone line to a central server. The clinician can request a review of the transmitted data at any time. Following FDA approval, we determined the usage and performance of the system.

**Results:**

Communicators (*n* = 53) were installed in 42 hospital facilities. The most common location was in the emergency department (*n* = 32, 60 %). There were 509 discreet transmissions, which were categorized as follows: no arrhythmia episodes in the past 72 h and no out of range measurements (*n* = 174, 34 %); arrhythmia episodes in past 72 h but no out of range measurements (*n* = 170, 33 %); and further review recommended (*n* = 130, 26 %). (In 35 [7 %] instances, interrogation without analysis was requested.) The further review interrogations were then sub-divided into those of a non-urgent and urgent nature. Overall, only 53 (10 %) of the 509 transmissions were classified as urgent. Clinicians had access to full technical consultation in ≤15 min in 89 % of instances.

**Conclusion:**

Our data demonstrate the feasibility of a new diagnosis-only, remote interrogation device and remote evaluation process for the interrogation of CIEDs in an in-hospital environment.

**Electronic supplementary material:**

The online version of this article (doi:10.1007/s10840-015-0091-4) contains supplementary material, which is available to authorized users.

Several hundred thousand patients undergo cardiac implantable electronic device (CIED) implantation in the USA annually [1, 2]. Recommendations for the routine follow-up of these devices, either through in-person evaluation or through remote monitoring, have recently been updated [1, 3]. However, patients often need device interrogation outside the elective environment to determine device settings, to assess the cause for an implantable cardioverter-defibrillator (ICD) shock, and/or to monitor surrogate markers of physiologic performance. Common scenarios include patients with a CIED presenting to the emergency department (ED) and those undergoing surgery or radiotherapy. In the case of the latter, device evaluation is often needed both immediately before and after intervention.

The responsibility for these non-elective device checks has fallen on industry-employed allied professionals (IEAPs) and/or trained physicians/allied health professionals (AHPs). However, unscheduled in-person interrogations impose a major challenge for workflow and come at the cost of delayed diagnosis and additional healthcare utilization. Furthermore, it has been previously shown that the vast majority of patients who present for device interrogation require no change to their medications or device reprogramming [4]. Given the development of remote monitoring technology and current clinical guidelines that recommend remote monitoring as the preferred method for routine CIED follow-up, we hypothesized that similar technology could be used and would be of value to interrogate devices quickly in an in-hospital environment and provide the clinician with a rapid initial review of findings. Thus, a diagnosis-only, remote interrogation device and remote evaluation process for CIEDs was developed to address this situation. To date, there have been no published reports of using remote monitoring to facilitate the triage of CIED patients who require device interrogation. The purpose of this study is to describe our initial clinical experience with this system.

## Methods

The LATITUDE Consult system provides access to expert technical review of data retrieved from an implanted Boston Scientific CIED without requiring on-site access to a Boston Scientific programmer or representative from Boston Scientific or trained healthcare professional (HCP) (e.g., AHP/physician) expert in CIED interrogation. This system requires minimal training for its use and is designed for use in locations such as the ED, radiation center, pre- and post-operative surgical units, hospital floors, and satellite clinics (including heart failure clinics).

**Fig. 1:**
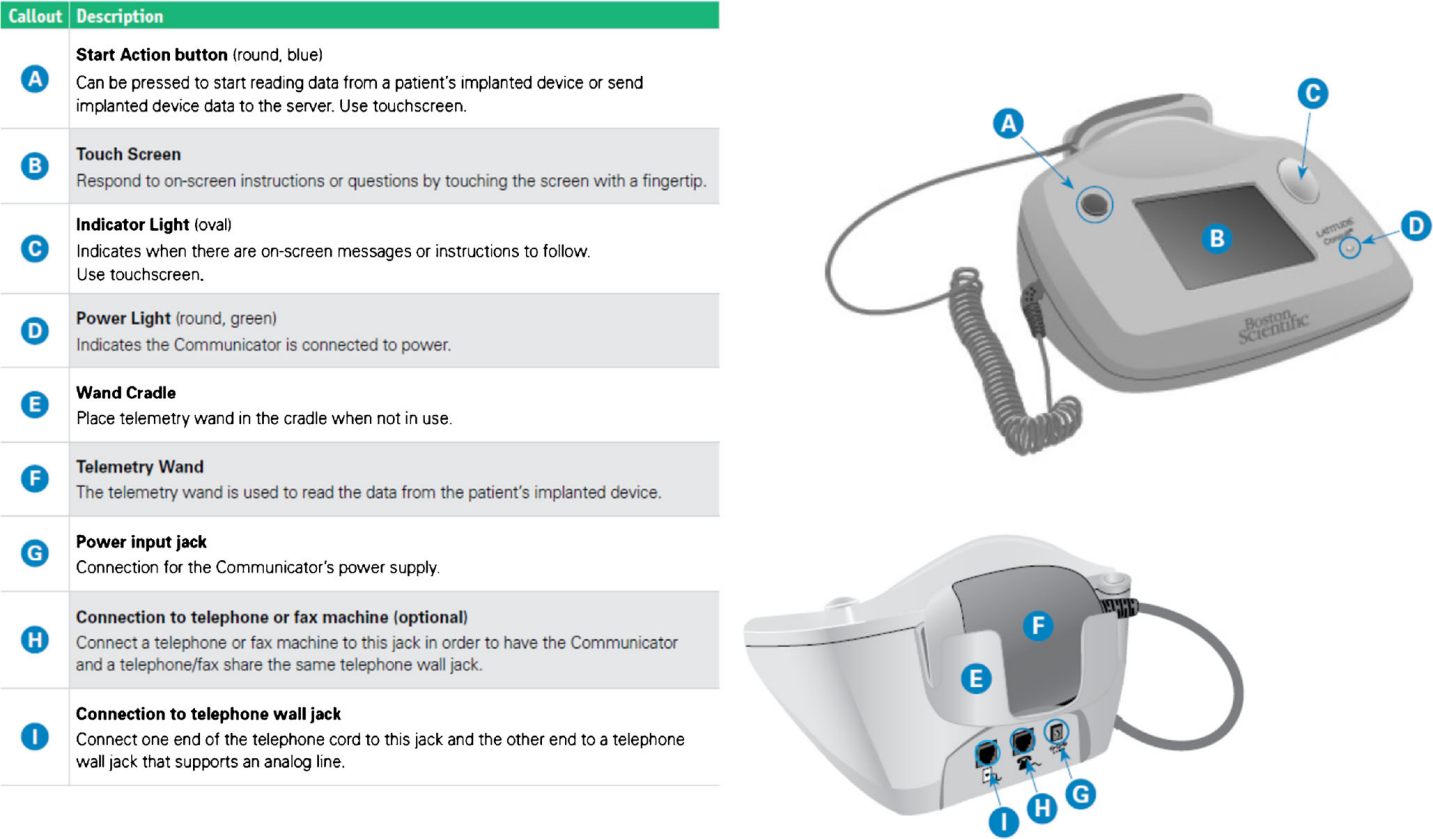
LATITUDE Consult Communicator

The LATITUDE Consult Communicator (Fig. 1) is a stand-alone interrogation device used to read the patient’s implanted device via a telemetry wand placed over the patient’s device. In this manner, it is possible to interrogate compatible permanent pacemakers (PPM), implantable cardioverter defibrillators (ICD), cardiac resynchronization therapy (CRT) pacemakers, and CRT defibrillators manufactured by Boston Scientific. The system can only read data; it cannot reprogram the device, perform commanded lead tests (such as pacing threshold tests or impedance measurements), or change any functions of the implanted device. Once retrieved from the device, upon command from the user, the data may be securely transmitted via an analog phone line to the Boston Scientific LATITUDE Server. For security, at the end of the process, the data are erased from the LATITUDE Consult Communicator itself.

**Fig. 2:**
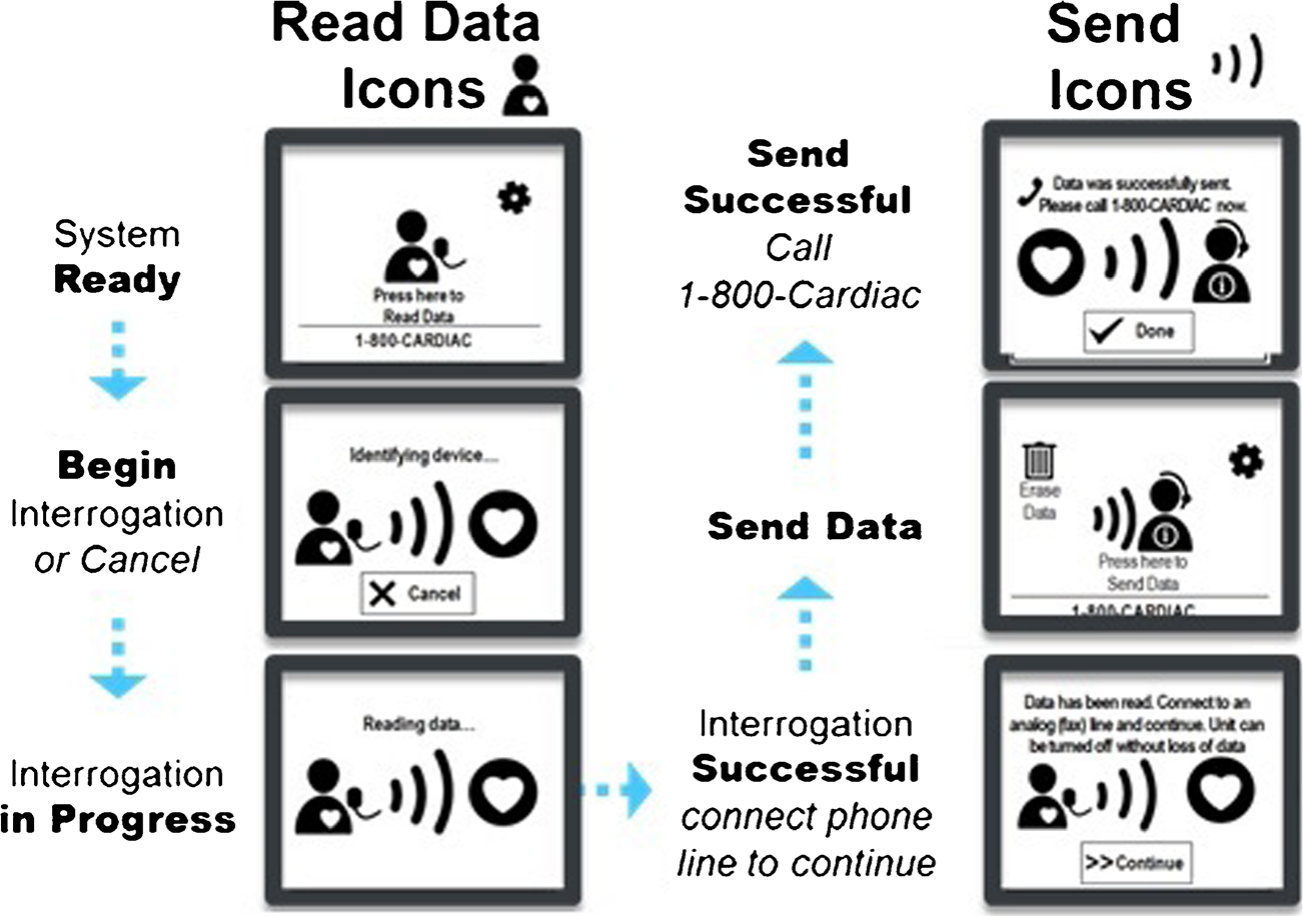
LATITUDE Consult Communicator screens providing status of the interrogation and data transmission process

To use the LATITUDE Consult system, the HCP or patient places the attached wand over the implanted device and presses the blue “Start Action” button. This begins the device interrogation process; the Communicator creates an inductive link with the implanted device and determines the model of the device. The stored data on the device are downloaded onto the Communicator internal storage (Table 1). On command by the user, by pressing the Continue/Send prompt on the LCD touchscreen, the data are securely transmitted via an analog phone line to Boston Scientific LATITUDE Consult Technical Services. The status of this process is pictorially shown via a series of LCD screens on the Communicator (Fig. 2) for the user.

**Table 1:** Device data transmitted for review during the LATITUDE Consult interrogation process

Downloaded data	Common values
Status messages	Status message displayed as applicable to indicate the current status of the device and/or leads as of the latest device transmission
Stored episodes	Stored episodes which occurred within the prior 72 h based on the type of device and programmed settings. Arrhythmia logbook contains all episodes available in the device at the time of upload
Battery status	Battery status as of the latest device transmission
Lead measurements	Most recent daily lead measurements for applicable chambers that are programmed on (e.g., intrinsic amplitude, pace impedance, pace threshold, shock impedance)
Trend graphs	Up to 12 months of all applicable leads, events, and health trends based on device type and programmed settings
Ventricular tachy counters	Counters for different types of tachy episodes based on the type of device and programmed settings
Pacing counters, histograms, and rate counts	Number of paced and sensed beats for applicable chambers (e.g., atrial, right ventricular, and left ventricular) since the last device reset and reset before the last in counter, histogram, and rate count formats
Device settings	Brady mode, pacing outputs, rate enhancements, and tachy therapy zones if applicable
Therapy mode, if applicable	Monitor, monitor + therapy, off
Presenting EGM	10 or 30 s of presenting intercardiac electrogram tracings based on the type of device

*tachy* tachycardia

After transmission of the implanted device data, the clinician can call LATITUDE Consult Technical Services 24 h a day, 7 days a week to request an expert technical review of the transmitted data and obtain a report of the interrogation. The clinician provides his/her name, the patient’s name, the model/serial number of the implanted device, the location of the interrogation, the reason for interrogation, and a phone number for an optional callback. The device data can be immediately reviewed by the Technical Services consultant, and the following reports can be sent via e-mail and/or fax for clinician review and entry into the patient’s medical records: (1) Transmission Report; (2) Quick Notes Report; (3) Combined Follow-up Report; (4) Presenting Electrogram Report; (5) Arrhythmia Logbook Report; (6) Event Detail/Episodes Report; and (7) Device Settings Report (Online Supplementary Material).

The clinician can discuss the reviewed implanted device data live with a trained US-based LATITUDE Consult Technical Services consultant either real-time with no need for a callback or after the LATITUDE Consult Technical Services representative performs a detailed review and calls back to discuss the data. Based on the findings of the interrogation, the local Boston Scientific technical representative can be contacted in order to provide additional consultation and/or perform reprogramming with the device programmer. Alternatively, for centers with regular usage of the LATITUDE Consult system, clinicians may opt to have device reports auto sent to a designated e-mail and/or fax number after the device data are successfully uploaded. In this scenario, the transmission report is sent to the clinician for review without analysis from a LATITUDE Consult Technical Services consultant.

All transmissions were cataloged into one of three categories based on the device data retrieved from the Consult transmission and discussion with the clinician. The first category was one with no device or arrhythmia concerns. The second category included patients who had an arrhythmia, who received therapy as programmed, and where the device was functioning appropriately. The final category included patients where further review of the CIED system was recommended either due to concerns about the device or when reprogramming was suggested to optimize device settings. These were further sub-classified into urgent and non-urgent transmissions. They were labeled urgent if the physician required immediate in-person analysis of the CIED by an IEAP or trained HCP with a programmer for possible device reprogramming. In non-urgent cases, the treating physician determined that further CIED follow-up was not necessary on an immediate basis and could wait for normal clinic hours in the event of an after-hours event or wait for a scheduled or walk-in appointment at the device clinic.

Following FDA approval of the LATITUDE Consult System in March 2014, a limited market release was performed with additional data recorded in order to evaluate the usage and performance of the system. The first Communicator was placed into service in May 2014; additional Communicators were brought online over the next year. Each Communicator had a discreet serial number, which allowed tracking of location from where each transmission emanated. We determined whether transmissions were occurring within business hours, defined as between 8 AM and 6 PM within that institution’s time zone from Monday–Friday, or outside business hours. Supplemental data on the performance of and reason for LATITUDE Consult uses was collected and logged into a Boston Scientific database for later analysis. The data collected are listed in Supplementary Table 1.

## Results

Ultimately, 53 unique Communicators were installed in 42 hospital facilities across the USA. We included facilities serving a variety of different populations in 20 states. In addition, of the 42 facilities involved in this study, 22 % were academic centers and 82 % were non-profit institutions. Seven (13 %) centers were located in an area with a population density of <100 persons per square mile, 20 (38 %) centers were located in an area with a population density between 100 and 999 persons per square mile, and 26 (49 %) centers were located in areas with a population density of >1000 persons per square mile. In any given facility, there were 1–4 Communicators stored in discreet locations. The three most common locations for the Communicators were in the ED (*n* = 32, 60 %), the post-anesthesia care unit (PACU; *n* = 8, 15 %), and a satellite clinic (*n* = 8, 15 %; Table 2). The median duration during which an individual center had access to the LATITUDE Consult Communicator was 4 months (interquartile range (IQR) 2, 7).

**Table 2:** Utilization of LATITUDE Consult Communicators stratified by location and indication for use

	Evaluation: patient symptoms	Post-procedure check	Evaluation: ICD shock	Other: only report requested	Pre-procedure check	Evaluation: non-device related	Evaluation: satellite clinic follow-up	Evaluation: allegation of beeping	Grand total
Emergency department (32 units)	181	5	83	15	2	6		2	294 (58 %)
PACU (8 units)	1	133	1	3	6	1			145 (28 %)
Satellite clinic (8 units)	13	2	1	7		1	4		28 (6 %)
Radiation therapy center (1 unit)		19	1		6				26 (5 %)
Hospital floor (3 units)	10			1		3			14 (3 %)
Other location (2 units)	1			1					2 (<1 %)
Total	206 (40 %)	159 (31 %)	86 (17 %)	27 (5 %)	14 (3 %)	11 (2 %)	4 (1 %)	2 (<1 %)	509

The first transmission was obtained in May 2014; the final transmission was received in March 2015. During this time period, there were 509 discreet transmissions analyzed from a LATITUDE Consult Communicator. The median number of transmissions from any center was 3 (IQR 2, 14). Transmissions most commonly originated from the ED (*n* = 294, 58 %) or the PACU (*n* = 145, 28 %; Table 2). The most common reasons for device check were for evaluation of patient symptoms, a post-procedure check, and for evaluation of a shock (Table 2). Transmissions were used to evaluate an ICD in 39 % of patients, a CRT-ICD in 30 % of patients, a PPM in 30 % of patients, and a CRT-PPM in 1 % of patients.

**Fig. 3:**
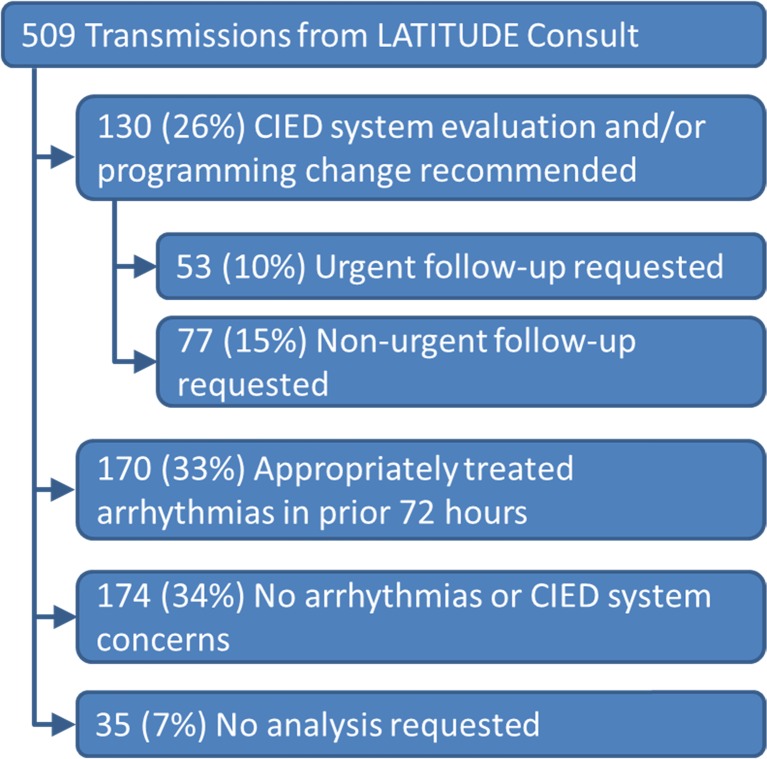
Categorization of all 509 LATITUDE Consult transmissions

The average number of transmissions per center was 9 (median = 3, IQR = 2–14). Transmissions were grouped into four categories based on the indication for device interrogation and analysis of the data, as shown in Fig. 3. Further review was recommended in 130 patients (see Table 3), in which the consulting physician requested immediate in-person analysis of the CIED by a trained IEAP or HCP with a programmer in 53 cases; the remaining 77 cases recommended analysis by an in-person IEAP or HCP with a programmer, but the analysis could wait until a scheduled or walk-in appointment. There were 170 transmissions in which arrhythmias occurred but were appropriately treated, and no further in-person analysis of the CIED was necessary. In 174 cases, no arrhythmia episodes were recorded in the CIED and no out-of-range measurements were observed which eliminated the need for an in-person evaluation of the device. In an additional 35 (7 %) instances, an interrogation only without analysis of the device data was requested. Overall, only 53 (10 %) of the 509 transmissions were classified as urgent; 51 (96 %) of these urgent transmissions originated from the ED.

**Table 3:** Interrogations requiring further review: characterization into non-urgent and urgent categories

Non-urgent (*n* = 77)	
Syncope but no episodes and no out-of-range measurements observed	22 (29 %)
Settings may be considered for programming optimization, e.g., PMT, RYTHMIQ, SBR, ATP, LV, sensors	18 (23 %)
Atrial arrhythmia episodes	16 (21 %)
New non-sustained episodes	12 (16 %)
Stored episodes older than 72 h are not uploaded by LATITUDE Consult, therefore not available for review	5 (6 %)
Low RV intrinsic amplitude or gradual rise in shock lead impedance	4 (5 %)
Urgent (*n* = 53)	
Multiple ATP and/or shocks delivered; therapy may be considered for optimization	18 (34 %)
Possible clinically inappropriate therapy; therapy may be considered for optimization	10 (19 %)
Rhythm detected in zone where therapy is not programmed; therapy may be considered for optimization	8 (15 %)
RV or LV intermittent capture or possible oversensing	7 (13 %)
Patient symptoms that may be arrhythmia related, e.g., PPM with tachycardia episodes, ICD with no therapy programmed	7 (13 %)
Possible battery depletion	3 (6 %)

*ATP* antitachycardia pacing, *ICD* implantable cardioverter-defibrillator, *LV* left ventricular, *PMT* pacemaker-mediated tachycardia, *PPM* permanent pacemaker, *RV* right ventricular, *SBR* sudden bradycardia response

Thus, for 90 % of all device interrogations, it was possible to triage patients as having either normal device function or findings that did not require immediate attention. This is significant from a workflow perspective as 154 (57 %) emergency department transmissions and 4 (29 %) of hospital floor transmissions occurred outside of traditional business hours; in contrast, transmissions from the PACU, radiation center, heart failure clinic. and satellite clinics largely originated during business hours, reflecting their usual hours of operation. The after-hours or weekend transmissions resulted in 25 urgent follow-up recommendations (24 in the emergency department and 1 on the hospital floor).

We assessed the efficiency of this approach to device interrogation during the initial 254 transmissions. In 130 (51 %) of these 254 transmissions, real-time review of the device interrogation was requested and provided. This means that the clinician stayed on the line with Boston Scientific Technical Services and thus had near instantaneous access to the results of the device interrogation. In the other 124 (49%) transmissions, the clinician requested a callback from Technical Services. This was achieved in 13.9 ± 6.3 min (range 2–44 min). In 89 % of instances, this was accomplished in 15 min or less. Delays in callback were accounted for either by the HCP being busy or by the presence of significant events that required extensive time for complete review. As an example, in the review that took 44 min, the patient had 9 episodes during which 16 shocks were delivered to the point that therapy had been exhausted.

## Discussion

This manuscript has several important findings. Foremost, for the first time, we have demonstrated the feasibility of a new diagnosis-only, remote interrogation device and remote evaluation process for the interrogation of all types of CIEDs (albeit from a single device manufacturer) in an in-hospital environment without the requirement of an on-site IEAP or trained physicians/AHPs. Second, the system could be utilized in a variety of settings both during and after traditional business hours. Importantly, in 89 % of instances, data analysis could be accomplished either in real time or within 15 min. Third, in 90 % of instances, the interrogation showed that no urgent device intervention was necessary for the management of the patient. This enables appropriate resources to efficiently be directed to the 10 % of patients with a significant finding on initial device interrogation.

Interrogation of CIEDs is essential to ensure the integrity of the hardware, to assess the adequacy of device programming relative to the patient’s clinical needs, and to monitor the physiologic and diagnostic data stored within the device. Historically, this has been accomplished through in-office device interrogations. However, more than a decade ago, technology became available that permitted remote follow-up of CIEDs from a patient’s home. Initially, guidelines recommended that device follow-up could be accomplished through in-office or remote interrogations [1]. With time, there was significant improvement in and acceptance of remote monitoring technology [5]. By reducing burdensome, non-actionable in-person office visits and streamlining workflow, remote monitoring of CIEDs can reduce the burden imposed by the “dramatic increase in the number of implants in the last decade [6, 7].” As a result, the most recent guidelines advocate the use of remote monitoring in preference to routine calendar-based in-office device interrogations [3].

However, to date, there has been little attention paid to the burden posed to patients, IEAPs, and HCPs when patients require unscheduled device interrogations in an in-hospital environment. Guidelines recommend that if an IEAP is requested to evaluate a pacemaker or ICD in the hospital, the physician who made the request should be immediately available by phone if he or she cannot provide direct supervision [8]. However, the guidelines are otherwise silent on the burden posed by these in-hospital device interrogations.

We hypothesized that modification of existing remote monitoring technology could be employed in an in-hospital environment. The Communicators used in this study were capable of interrogating most Boston Scientific CIEDs, irrespective of device type. Once the HCP initiated the interrogation, a report was provided similar to what would be available were the device interrogated using a programmer. Depending on the preference of the HCP, an interpretation of the interrogation could be left to the provider or be performed by a trained Boston Scientific employed technician. Irrespective, data were available quickly (most commonly within 15 min) allowing patients to be triaged in an efficient manner.

This has particular importance in settings like the ED where capacity and throughput are continually challenged [9, 10]. The ED is frequently the site for unscheduled device checks. As an example, 83 (97 %) of the 86 transmissions in this study to evaluate an ICD shock were initiated in the ED. In 76 % of these instances, transmissions occurred outside of traditional business hours. Even in this cohort, it was possible to determine quickly and efficiently that in 60 % of patients, no further immediate device-related intervention was necessary.

Another important utilization of the system is pre- and post-intervention when interrogations are necessary (e.g., administration of radiotherapy, following a surgical procedure using electrocautery) to ensure adequate functioning of the CIED [11, 12]. There were 171 such transmissions in this study. It was determined that no further immediate device-related intervention was necessary in 170 (>99 %) of these instances. Prior to the availability of this system, a patient would have to wait pre- and post-procedure for someone to interrogate the device. Alternatively, an IEAP performed the pre-procedure device interrogation and then waited for the procedure to complete to perform a post-procedure interrogation. In addition to the inconvenience to patients, this results in tremendous waste of time and resources, especially given that almost all patients have normally functioning devices.

### Limitations

This manuscript has a few limitations. First, once the system was installed in any location within a participating hospital, all subsequent interrogations of a compatible Boston Scientific pacemaker or defibrillator at that location were intended to be performed using this remote interrogation system. However, there is no way to exclude entirely that an IEAP or other HCPs performed a device interrogation using a standard device programmer for initial device evaluation during this time period. Second, as this was a pilot study, we did not have a comparative group of patients who underwent device interrogations using a customary programmer-based approach. This would have occurred when a device interrogation occurred in a hospital location where there was no communicator.

### Conclusions

Our data suggest that interrogation of CIEDs coupled to a remote server in various hospital-based clinical settings is clinically feasible and can be performed quickly and efficiently. Based on clinical experience, it is self-evident that device interrogation could not have been achieved in a similar time frame had we relied on the usual practice of using an IEAP or trained physicians/AHP. However, in the future, formal cost-effectiveness analysis comparing this approach with the usual IEAP/HCP programmer-based device interrogations in an in-hospital environment can be considered. The availability of data transfer using Wi-Fi connectivity as well as directly over the cellular network will likely enhance the ability of this technology to operate efficiently irrespective of the local hospital environment. Future efforts should be directed towards using this type of technology routinely when CIED patients require device evaluation in an in-hospital environment.

## Electronic supplementary material

Below is the link to the electronic supplementary material.

Online Supplemental Material 1 Examples of available reports following transmission from the LATITUDE Consult system. The patient presented to the Emergency Department for evaluation after receiving an ICD shock. (PDF 744 kb)

## Abbreviations

AHP: allied health professional
CIED: cardiac implantable electronic device
CRT: cardiac resynchronization therapy
ED: emergency department
FDA: Food and Drug Administration
HCP: healthcare professional
ICD: implantable cardioverter-defibrillator
IEAP: industry-employed allied professional
IQR: interquartile range
PACU: post-anesthesia care unit
PPM: permanent pacemaker
