# Supplementary material for: Performance of a remote interrogation system for the in-hospital evaluation of cardiac implantable electronic devices
Source: J Interv Card Electrophysiol. 2015 Dec 22;46:121–8. doi: 10.1007/s10840-015-0091-4 (PMC4923098; doi:10.1007/s10840-015-0091-4)
Supplement: Supplementary file 1 — Examples of available reports following transmission from the LATITUDE Consult system. The patient presented to the Emergency Department for evaluation after receiving an ICD shock. (PDF 744 kb) [file 10840_2015_91_MOESM1_ESM.pdf]

Transmission Report

Boston Scientific

[redacted]

Date of Birth: [redacted]
Device: **E110/[redacted] Defibrillator**
Communicator Model/SN: **6299/[redacted]**

Summary

Reviewed As:	Implanted device has recorded episodes in past 72 hrs. No out of range measurements observed.
Notes:	Patient name: [redacted] Anti-Tachycardia Pacing and Shock(s) delivered in past 72 hrs. Detected in VF zone, rate >200 bpm. Rhythm converted. Normal follow-up recommended with device following physician.
Interrogation Date:	Aug [redacted], 2014 01:17 EDT
Transmission Date:	Aug [redacted], 2014 00:21 EDT

Contact Information

Treating Clinician:	Dr. [redacted]
Reason For Transmission:	Evaluation: report of shock
Location Name:	[redacted] REGIONAL MEDICAL CENTER
Location Type:	ER
Caller Name:	Dr. [redacted]
Caller Phone:	[redacted]

Registered Information

Communicator Model/SN:	6299/[redacted]
Location Name:	[redacted] REGIONAL MEDICAL CENTER
Location Type:	ER
Address:	[redacted]
Phone:	[redacted]

Disclaimer

- The Boston Scientific Transmission Report is based solely upon a review of the reports transmitted at a specific point in time by the LATITUDE Consult™ Communicator at the point of care facility, which can include up to 12 months of device/lead trend data and most recent 72 hours of stored episodes prior to interrogation.
- The Boston Scientific Transmission Report is focused on information generated by the implanted system and is intended to convey status of the implanted system. It is not a patient diagnosis. The report should not be considered as medical advice, and the attending health care professional is solely responsible for the degree of care, skill, adequacy, effectiveness and manner of use of this report in the care and treatment of patients. Boston Scientific is not a provider of patient health care services by virtue of the provision of this report.
- A transmission review of "Further review recommended" indicates that further review may be appropriate, which may include consulting with the patient's cardiac physician and/or interrogating the device with a Boston Scientific programmer to enable further analysis.
- This report contains legally privileged health information and is solely intended for use by authorized recipients at the associated account in furtherance of treatment of their patient. If you are not the intended recipient, please be advised that any disclosure, copying, distribution, or use of the content of this report is strictly prohibited. If you have received or are accessing this report in error, please notify Boston Scientific immediately at 1-800-CARDIAC (227-3422) and destroy any copies.

©2013 Boston Scientific Corporation or its affiliates. All rights reserved.

Quick Notes Report

LATITUDE Consult™ System - Quick Notes Report | Report Created: Aug [redacted], 2014

[redacted]

Date of Birth: [redacted]
Device: **TELIGEN 100 E110/[redacted]**
Clinic: [redacted] **REGIONAL MEDICAL CE**
Tachy Mode: **Monitor + Therapy**

Latest Device Transmission: **Aug [redacted], 2014 01:17 EDT**
Last Office Interrogation: **May [redacted], 2014**
Implant Date: [redacted]**, 2012**

Status Messages (Aug [redacted], 2014 01:17 EDT)

⚠ Ventricular shock therapy delivered to convert arrhythmia.

Events Since Last Reset (May [redacted], 2014)

Jul 31, 2014 20:22	⚠	VF at 233 bpm, ATPx1, 41J
Jul 30, 2014 18:05		VF at 224 bpm, ATPx1

Battery OK

Approximate time to explant: **10.5 years** from Aug [redacted], 2014

Last Capacitor Re-form	Jul 31, 2014 20:22
Charge Time	8.9 s

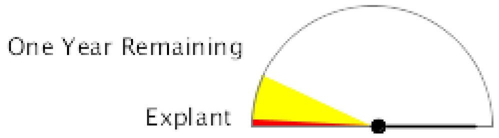

Leads

	Implant ([redacted], 2012)	Most Recent In-Office Measurement (May [redacted], 2014)	Most Recent Daily Measurement (Jul [redacted], 2014)
**Atrial**			
Intrinsic Amplitude	1.0 mV	1.6 mV	2.2 mV
Pace Impedance	400 Ω	323 Ω	341 Ω
Pace Threshold	0.6 V @ 0.5 ms	1.4 V @ 0.5 ms	
**Ventricular**			
Intrinsic Amplitude	>25.0 mV	>25.0 mV	>25.0 mV
Pace Impedance	550 Ω	554 Ω	572 Ω
Pace Threshold	0.5 V @ 0.5 ms	0.7 V @ 0.5 ms	
**Shock**			
Shock Impedance	60 Ω	79 Ω	80 Ω

Brady Counters	Since Last Reset May [redacted], 2014
Atrial	0 % Paced
Ventricular	1 % Paced

Settings

Ventricular Tachy

VF	200 bpm	ATP		41 J, 41 J, 41 Jx6
VT	170 bpm	Scan	ATP2 Off	41 J, 41 J, 41 Jx4

Atrial Tachy

ATR Mode Switch	170 bpm	DDIR

Brady

Mode	DDD
Lower Rate Limit	40 ppm
Maximum Tracking Rate	130 ppm
Maximum Sensor Rate	130 ppm
Paced AV Delay	280 - 300 ms
Sensed AV Delay	280 - 300 ms
A-Refractory (PVARP)	180 - 280 ms
V-Refractory (VRP)	230 - 250 ms

Pacing Output

Atrial	3.0 V @ 0.5 ms
Ventricular	2.0 V @ 0.5 ms

Sensitivity

Atrial	AGC 0.25 mV
Ventricular	AGC 0.6 mV

Leads Configuration (Pace/Sense)

Atrial	Bipolar
Ventricular	Bipolar

Sensor

Accelerometer	ATR Only

©2013 Boston Scientific Corporation or its affiliates. All rights reserved.

Clinician Signature

Combined Follow-Up Report

[redacted]

Date of Birth: [redacted]
Device: **TELIGEN 100 E110/[redacted]**
Clinic: [redacted] **REGIONAL MEDICAL CE**
Tachy Mode: **Monitor + Therapy**

Latest Device Transmission: **Aug [redacted], 2014 01:17 EDT**
Last Office Interrogation: **May [redacted], 2014**
Implant Date: [redacted]**, 2012**

Status Messages (Aug [redacted], 2014 01:17 EDT)

⚠ Ventricular shock therapy delivered to convert arrhythmia.

Events Since Last Reset (May [redacted], 2014)

Jul 31, 2014 20:22	⚠	VF at 233 bpm, ATPx1, 41J
Jul 30, 2014 18:05		VF at 224 bpm, ATPx1

Battery OK

Approximate time to explant: **10.5 years** from Aug [redacted], 2014	
Last Capacitor Re-form	Jul 31, 2014 20:22
Charge Time	8.9 s

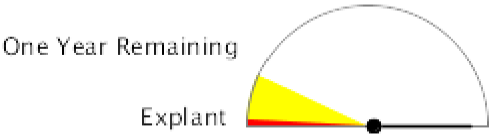

See last page for additional battery information.

Leads	Implant ([redacted] 2012)	Most Recent In-Office Measurement (May [redacted], 2014)	Most Recent Daily Measurement (Jul [redacted], 2014)
**Atrial**			
Intrinsic Amplitude	1.0 mV	1.6 mV	2.2 mV
Pace Impedance	400 Ω	323 Ω	341 Ω
Pace Threshold	0.6 V @ 0.5 ms	1.4 V @ 0.5 ms	
**Ventricular**			
Intrinsic Amplitude	>25.0 mV	>25.0 mV	>25.0 mV
Pace Impedance	550 Ω	554 Ω	572 Ω
Pace Threshold	0.5 V @ 0.5 ms	0.7 V @ 0.5 ms	
**Shock**			
Shock Impedance	60 Ω	79 Ω	80 Ω

Settings

Ventricular Tachy

VF	200 bpm	ATP		41 J, 41 J, 41 Jx6
VT	170 bpm	Scan	ATP2 Off	41 J, 41 J, 41 Jx4

Atrial Tachy

ATR Mode Switch	170 bpm	DDIR

Brady

Mode	DDD
Lower Rate Limit	40 ppm
Maximum Tracking Rate	130 ppm
Maximum Sensor Rate	130 ppm
Paced AV Delay	280 - 300 ms
Sensed AV Delay	280 - 300 ms
A-Refractory (PVARP)	180 - 280 ms
V-Refractory (VRP)	230 - 250 ms

Pacing Output

Atrial	3.0 V @ 0.5 ms
Ventricular	2.0 V @ 0.5 ms

Sensitivity

Atrial	AGC 0.25 mV
Ventricular	AGC 0.6 mV

Leads Configuration (Pace/Sense)

Atrial	Bipolar
Ventricular	Bipolar

Sensor

Accelerometer	ATR Only

Rhythm ID Update

Last Successful Completion	Jul [redacted], 2014 22:56

©2013 Boston Scientific Corporation or its affiliates. All rights reserved.

Clinician Signature

Trend Graphs

Most Recent Measurement: **Jul [redacted], 2014**

Heart Rate

Max
Mean
Min

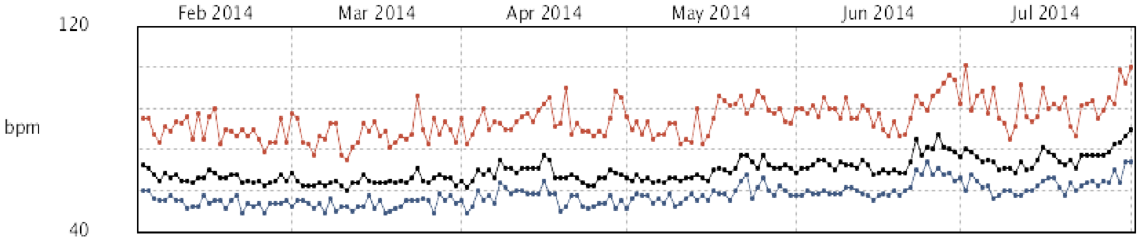

Respiratory Rate

Max
Median
Min

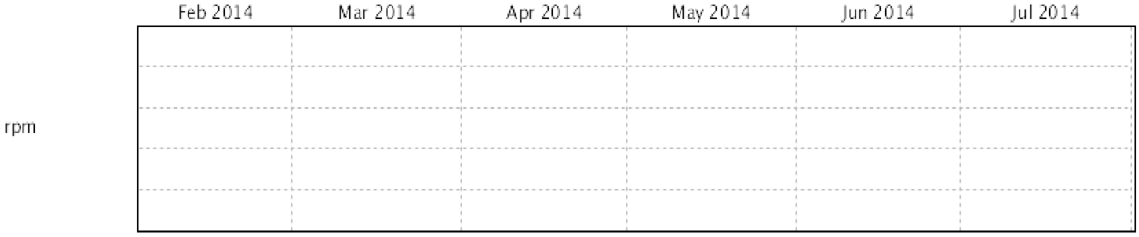

Activity Level

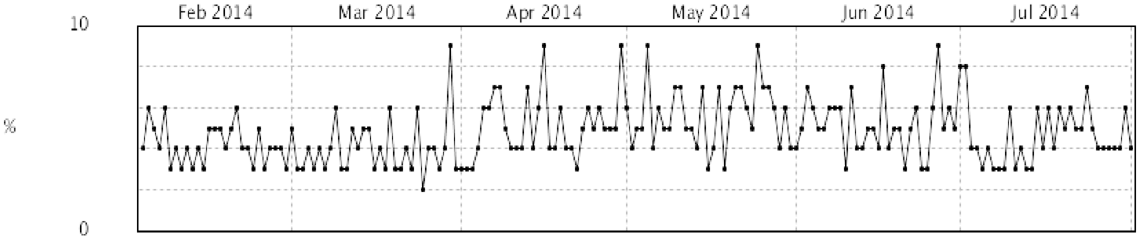

Atrial Burden

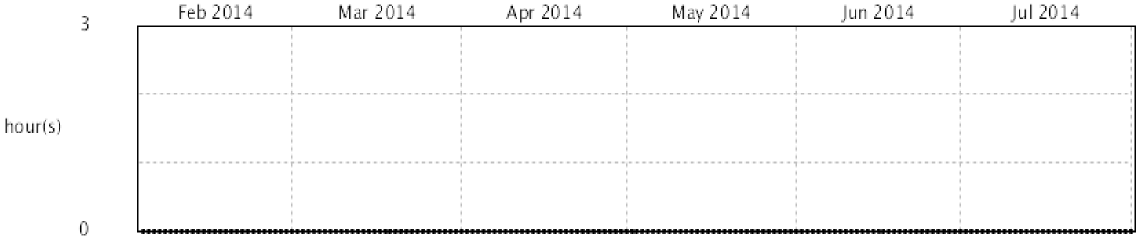

Atrial

Intrinsic Amplitude

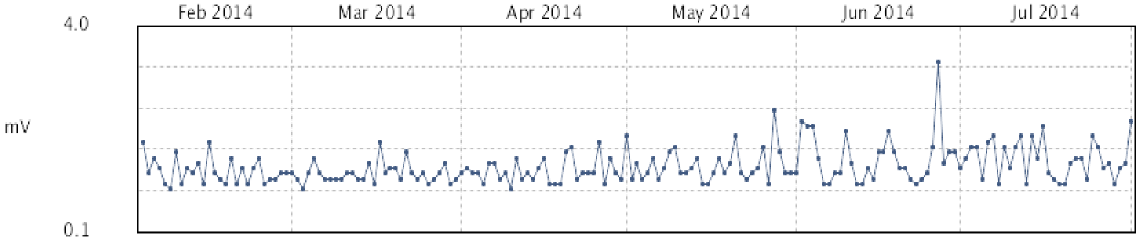

Atrial

Pace Impedance

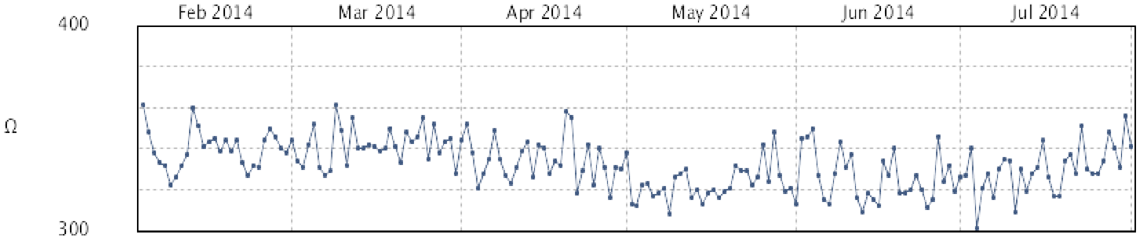

Ventricular

Intrinsic Amplitude

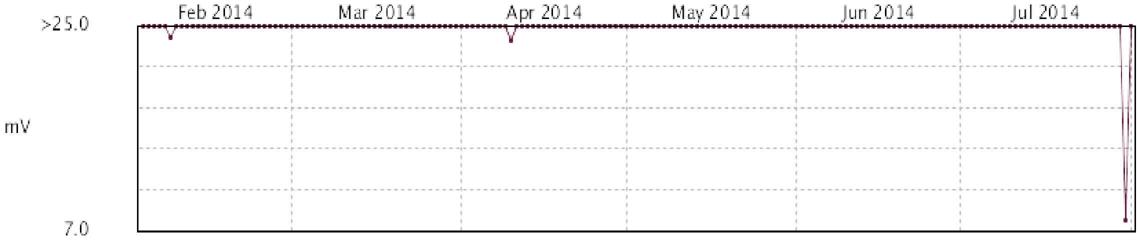

Ventricular

Pace Impedance

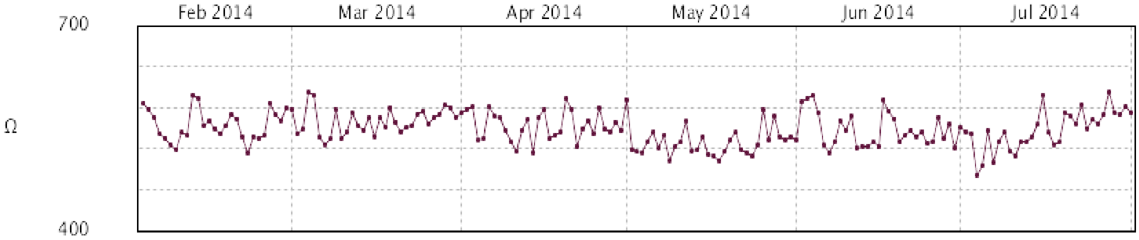

©2013 Boston Scientific Corporation or its affiliates. All rights reserved.

Shock

Shock Impedance

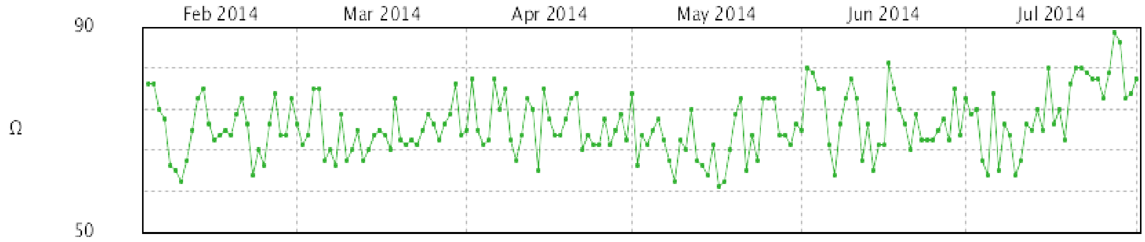

Ventricular Tachy Counters	Since Last Reset May [redacted], 2014	Device Totals
**Ventricular Episode Counters**		
Total Episodes	2	6
**Treated**		
⚠ VF Therapy	2	3
VT Therapy	0	0
VT-1 Therapy	0	0
Commanded Therapy	0	0
**Non-Treated**		
No Therapy Programmed	0	0
NonsustainedV Episodes	0	3
Other Untreated Episodes	0	0
**Ventricular Therapy Counters**		
ATP Delivered	2	3
ATP % Successful	50	33
Shocks Delivered	1	3
First Shock % Successful	100	50
Shocks Diverted	1	1

Brady Counters	Reset Before Last Sep [redacted], 2013	Since Last Reset May [redacted], 2014
**Counters**		
% A Paced	0	0
% V Paced	7	1
**Intrinsic Promotion**		
AV Search		
% Successful	0	0
Rate Hysteresis		
% Successful	0	0
**Atrial Burden**		
Episodes by Duration		
< 1 minute	0	0
1 min - < 1 hr	0	0
1 hr - < 24 hr	0	0
24 hr - < 48 hr	0	0
> 48 hr	0	0
Total PACs	2223	1123
**Ventricular Counters**		
Total PVCs	216428	188778
Three or More PVCs	0	0

©2013 Boston Scientific Corporation or its affiliates. All rights reserved.

Histograms

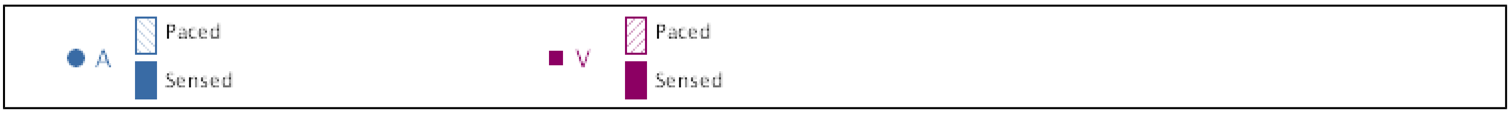

Reset Before Last	Since Last Reset
Sep [redacted], 2013 to May [redacted], 2014	May [redacted], 2014 to Aug [redacted], 2014

Atrial Activity

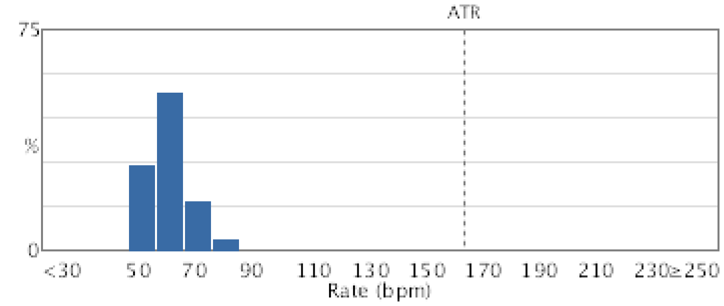

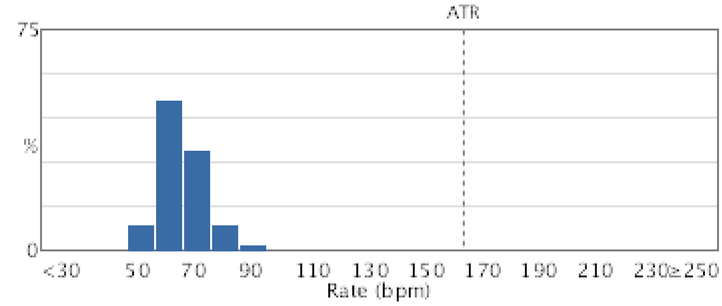

Ventricular Activity

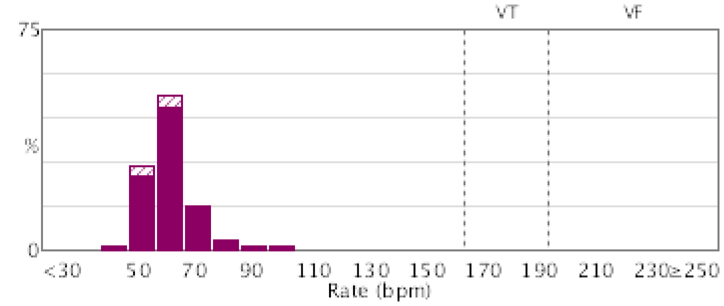

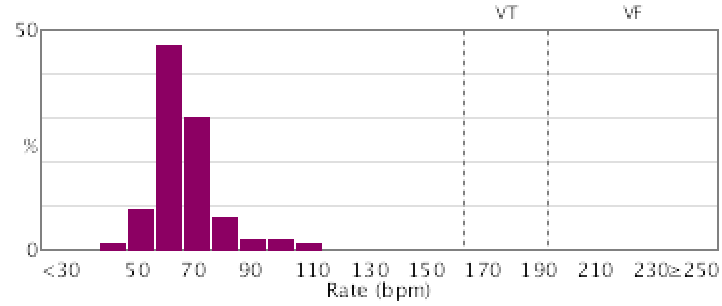

Ventricular Response to Atrial Pace

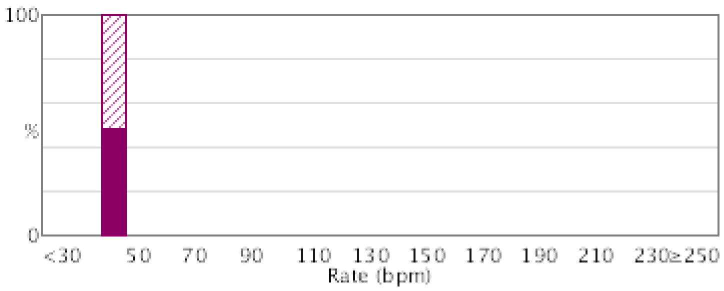

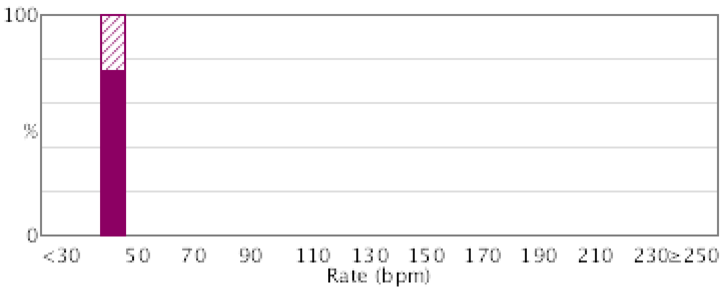

Ventricular Response to Atrial Sense

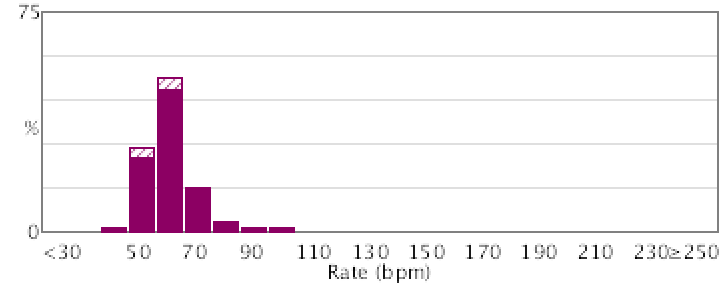

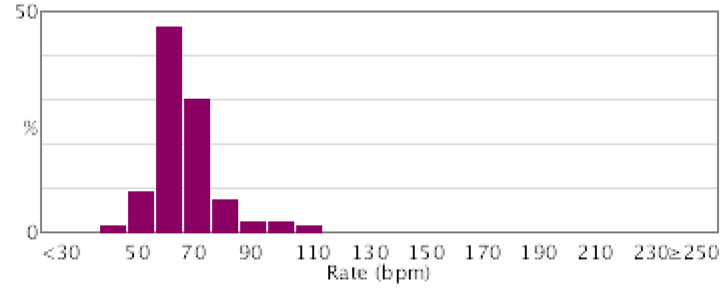

©2013 Boston Scientific Corporation or its affiliates. All rights reserved.

Histogram Rate Counts

Atrial	Reset Before Last Sep [redacted], 2013 to May [redacted], 2014		Since Last Reset May [redacted], 2014 to Aug [redacted], 2014	
Rate (bpm)	Paced	Sensed	Paced	Sensed
0-29	0	2	0	0
30-39	0	0	0	3
40-49	31	91.1K	1	824
50-59	0	6.6M	0	543.4K
60-69	0	12.4M	0	3.5M
70-79	0	3.7M	0	2.3M
80-89	0	604.3K	0	528.9K
90-99	22	98.0K	0	91.2K
100-109	0	16.8K	0	12.2K
110-119	0	3.7K	0	609
120-129	0	184	0	113
130-139	0	111	0	79
140-149	0	64	0	36
150-159	0	45	0	16
160-169	0	29	0	17
170-179	0	17	0	7
180-189	0	15	0	8
190-199	0	6	0	5
200-209	0	16	0	4
210-219	0	11	0	5
220-229	0	11	0	2
230-239	0	12	0	3
240-249	0	7	0	1
>=250	0	840	0	432

Ventricular	Reset Before Last Sep [redacted], 2013 to May [redacted], 2014		Since Last Reset May [redacted], 2014 to Aug [redacted], 2014	
Rate (bpm)	Paced	Sensed	Paced	Sensed
0-29	0	0	0	0
30-39	2	26	0	0
40-49	19.7K	242.6K	1.8K	91.8K
50-59	641.2K	6.0M	8.4K	632.3K
60-69	909.0K	11.2M	21.2K	3.3M
70-79	84.0K	3.5M	2.1K	2.1M
80-89	2.9K	625.9K	2.3K	489.5K
90-99	668	202.7K	1.7K	136.9K
100-109	388	119.9K	1.3K	161.1K
110-119	211	27.8K	863	54.9K
120-129	17	8.9K	20	17.4K
130-139	0	4.3K	0	8.3K
140-149	0	1.6K	0	3.2K
150-159	0	399	0	652
160-169	0	125	0	137
170-179	0	36	0	55
180-189	0	5	0	22
190-199	0	2	0	6
200-209	0	0	0	3
210-219	0	0	0	6
220-229	0	0	0	38
230-239	0	0	0	10
240-249	0	0	0	0
>=250	0	0	14	0

©2013 Boston Scientific Corporation or its affiliates. All rights reserved.

Presenting EGM (Aug [redacted], 2014)

EGM displayed at 25mm per second

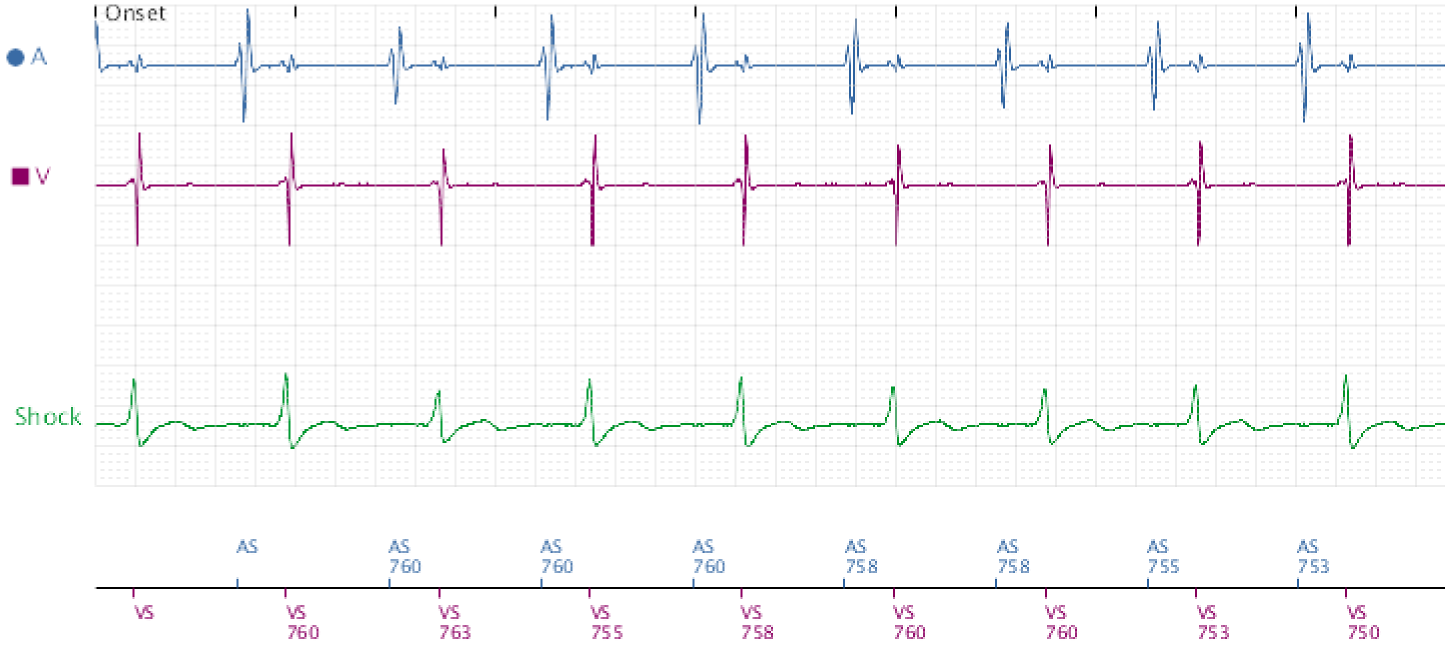

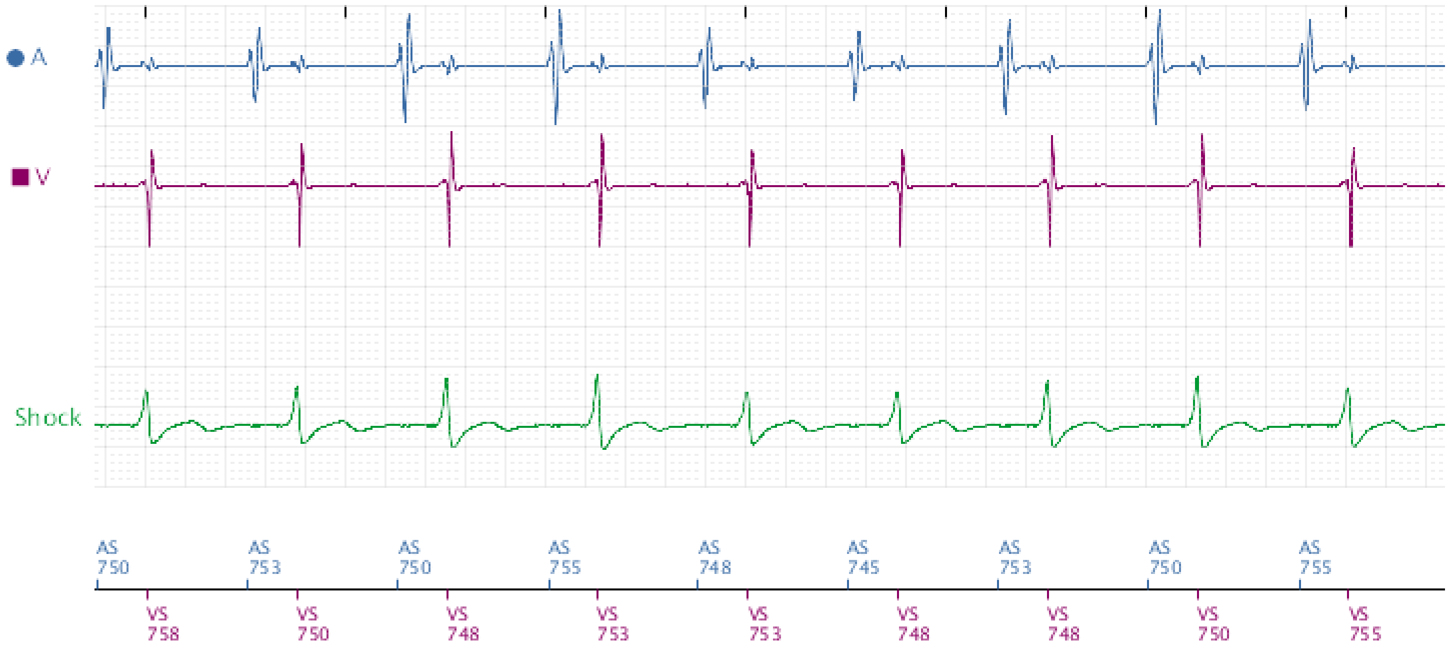

©2013 Boston Scientific Corporation or its affiliates. All rights reserved.

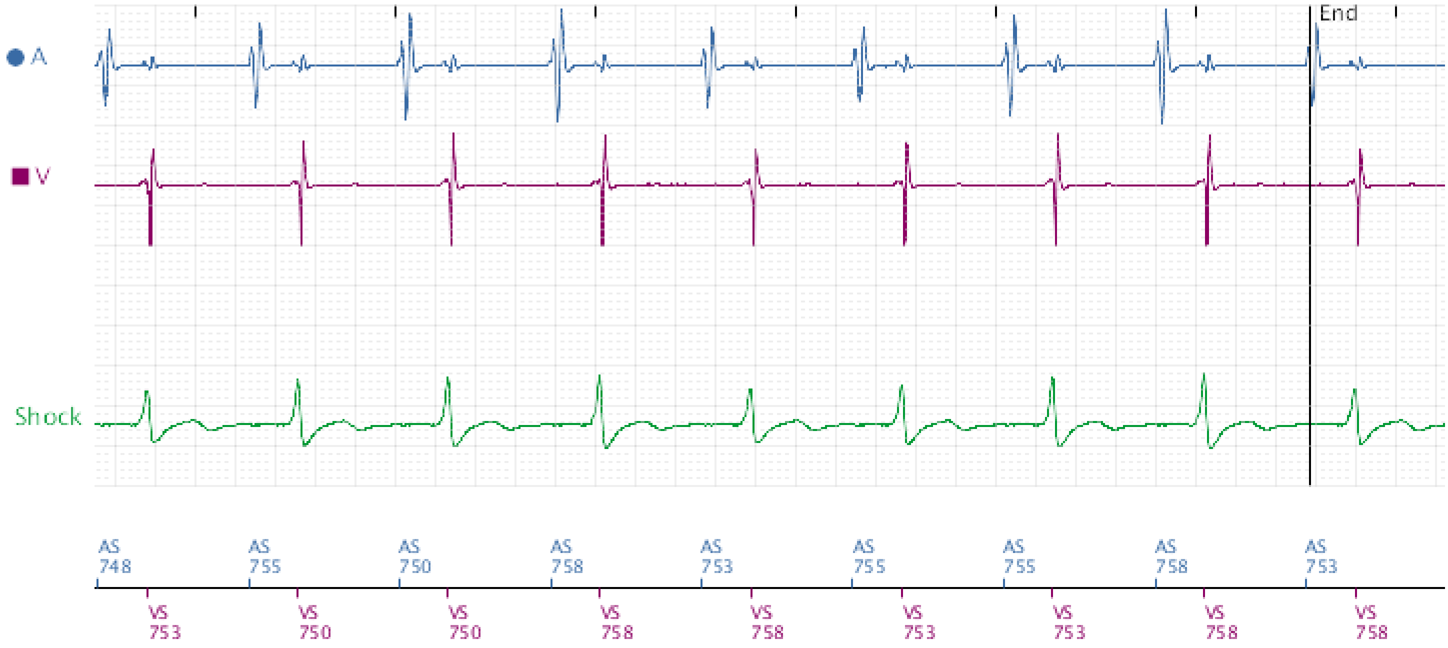

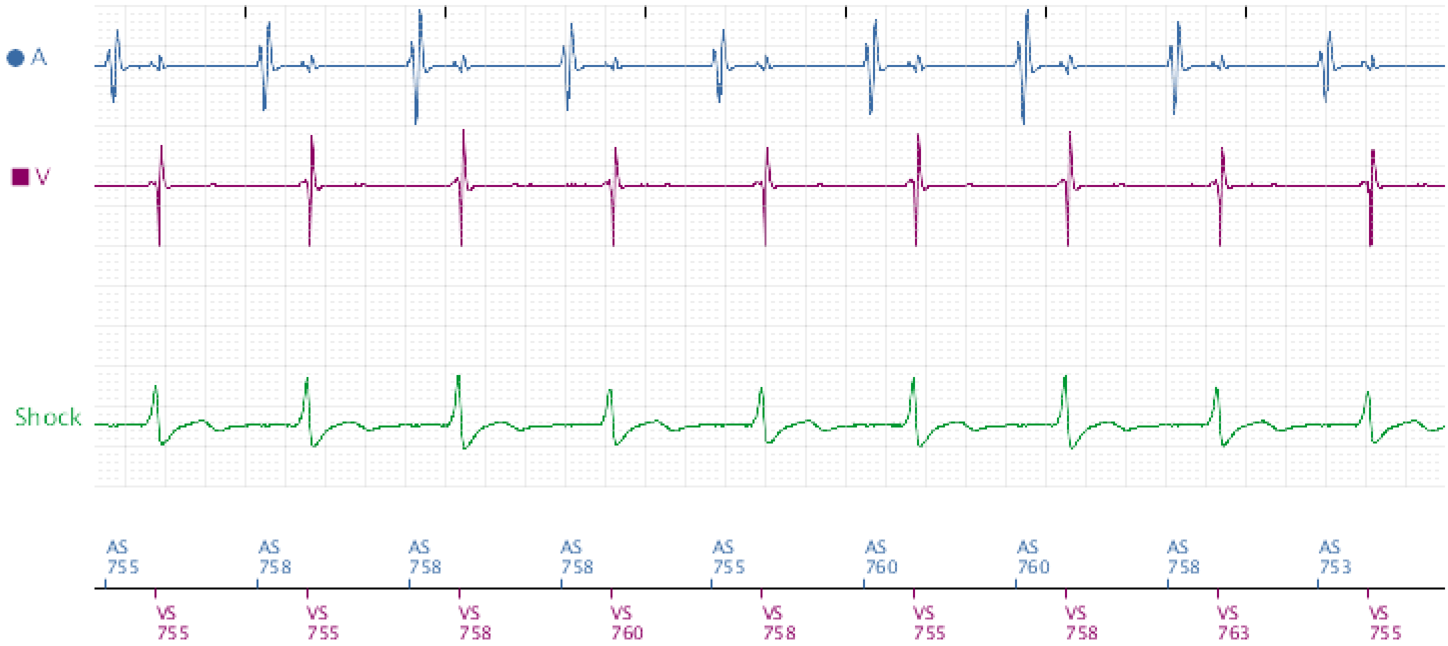

©2013 Boston Scientific Corporation or its affiliates. All rights reserved.

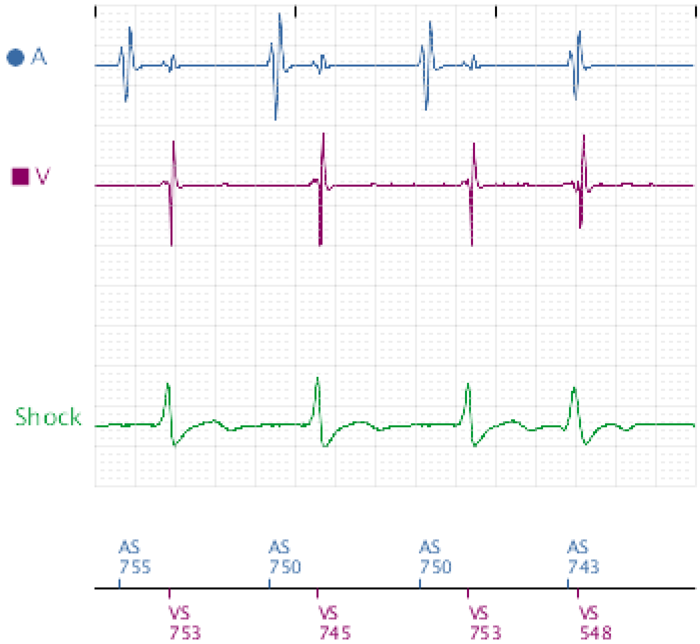

Battery (Continued)

Last Delivered Shock	Jul 31, 2014 20:22
Energy	41 J
Charge Time	8.9 s
Shock Impedance	68 Ω
Beep when Explant is Indicated	On

Time Since Implant	22 months
Charge Remaining	1.52 ampere-hours
Power Consumption	35 µW

(Measured with programmed parameters)

This device is using 78% of the power it would use at the following parameters:

A	100% pacing, 60 ppm, 2.5 V, 0.4 ms, 500 Ω
V	100% pacing, 60 ppm, 2.5 V, 0.4 ms, 700 Ω

©2013 Boston Scientific Corporation or its affiliates. All rights reserved.

Presenting Electrogram Report

Boston Scientific

[redacted]

Date of Birth: [redacted]
Device: **TELIGEN 100 E110/[redacted]**
Clinic: [redacted] **REGIONAL MEDICAL CE**
Tachy Mode: **Monitor + Therapy**

Latest Device Transmission: **Aug [redacted], 2014 01:17 EDT**
Last Office Interrogation: **May [redacted], 2014**
Implant Date: [redacted] **2012**

Presenting EGM (Aug [redacted], 2014)

EGM displayed at 25mm per second

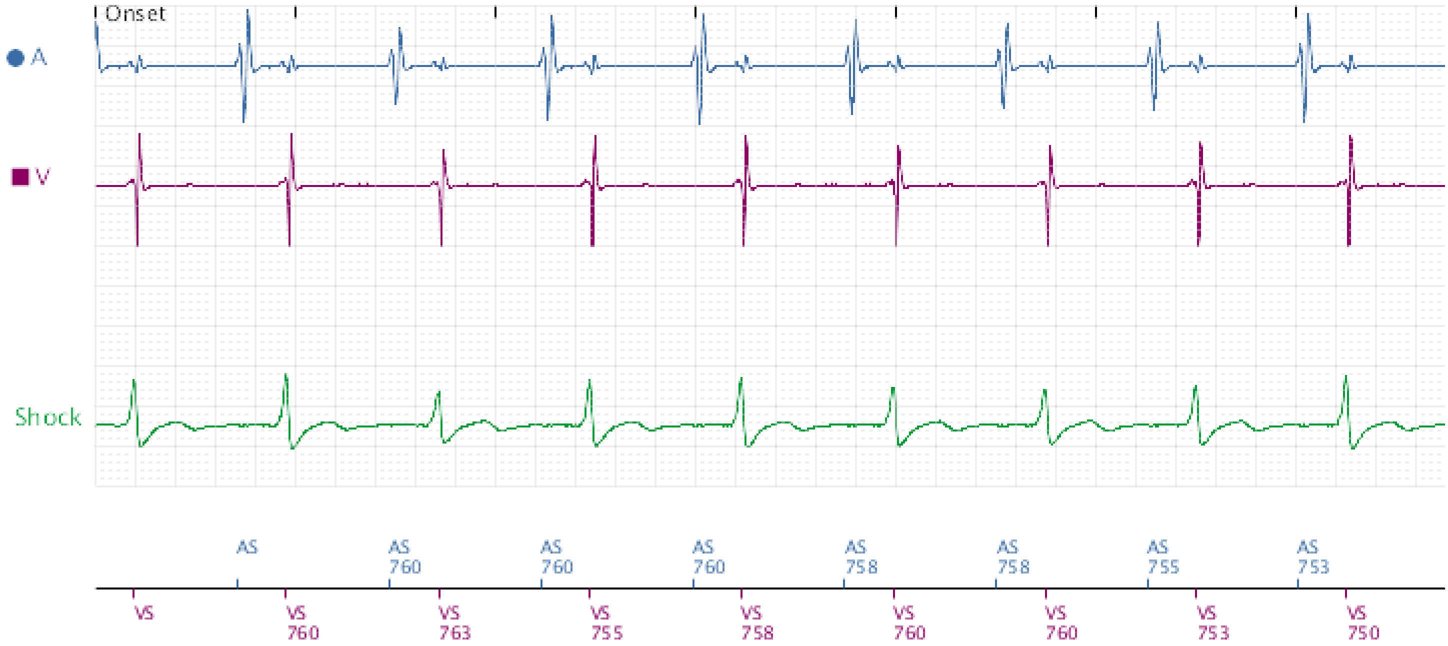

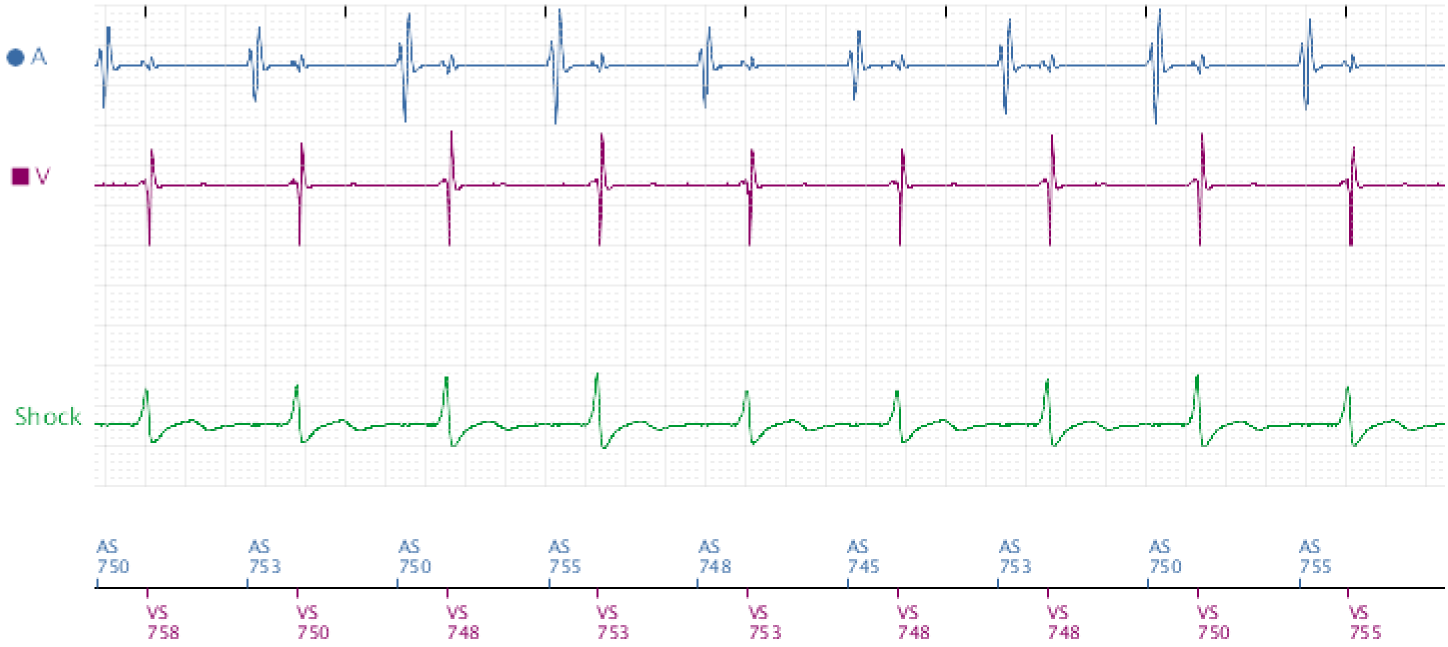

©2013 Boston Scientific Corporation or its affiliates. All rights reserved.

Clinician Signature

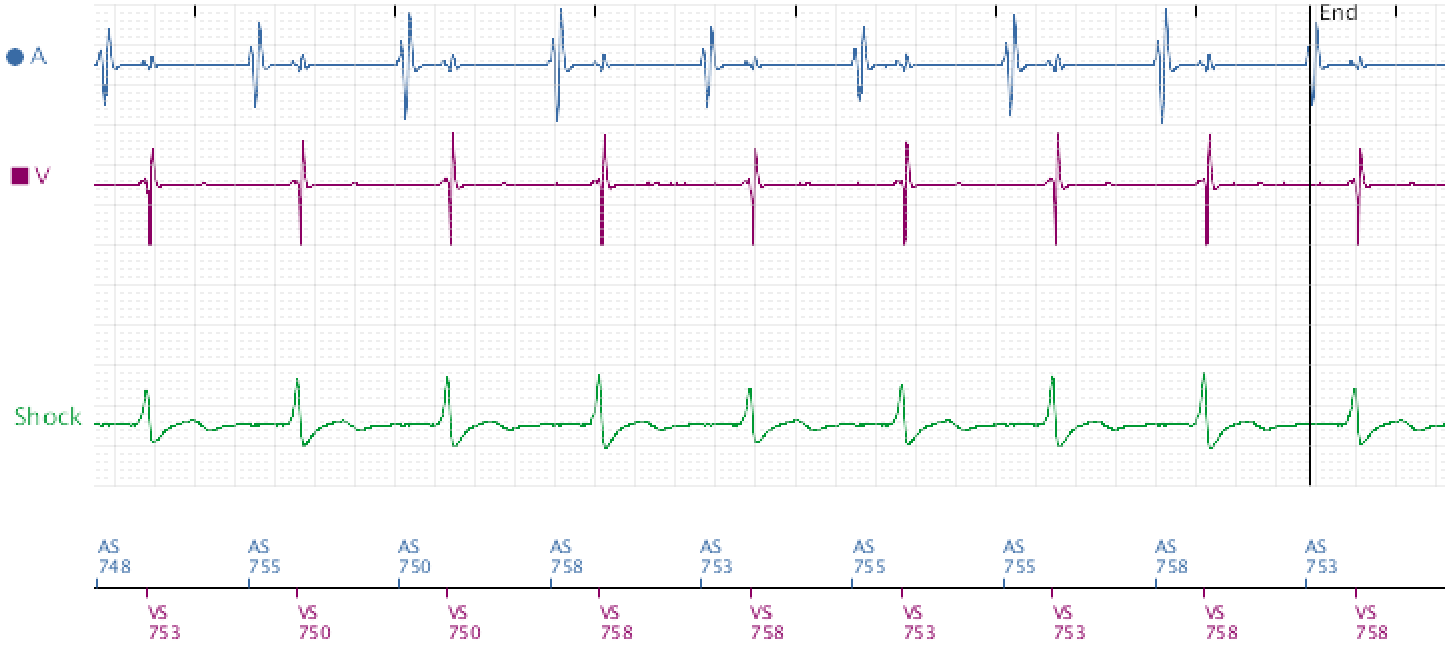

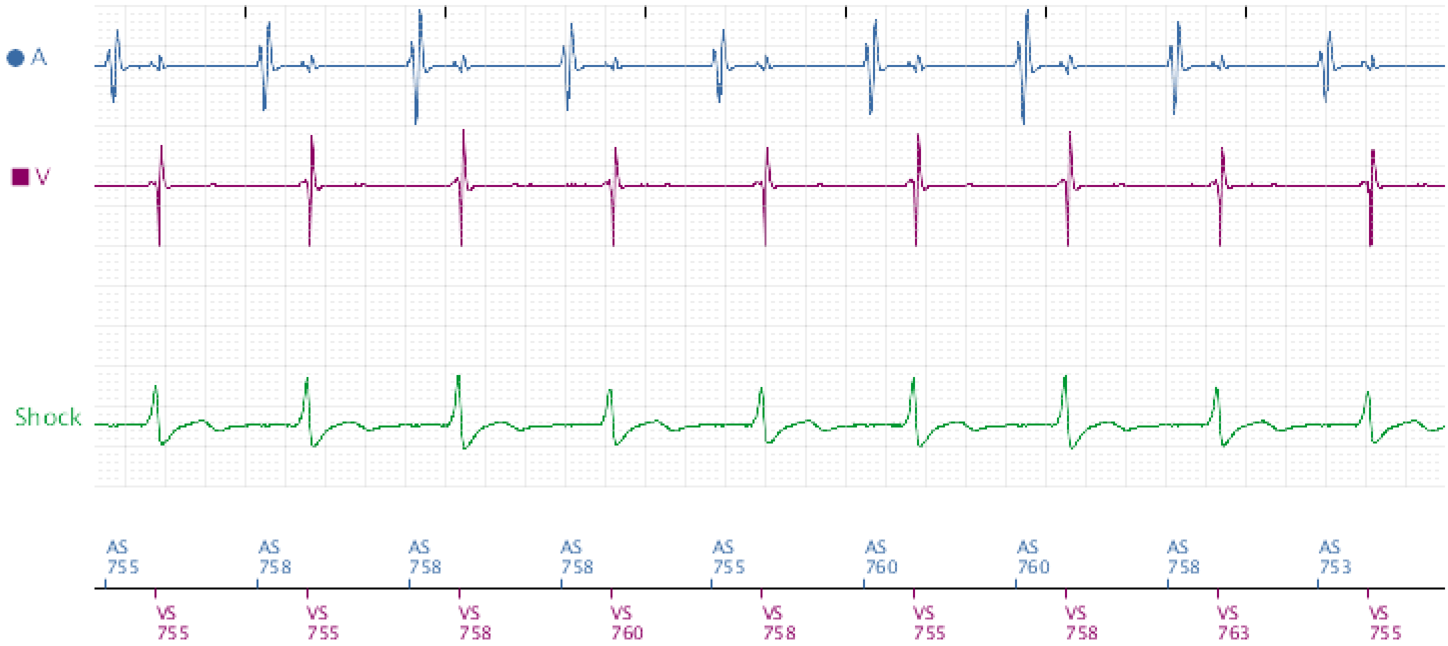

©2013 Boston Scientific Corporation or its affiliates. All rights reserved.

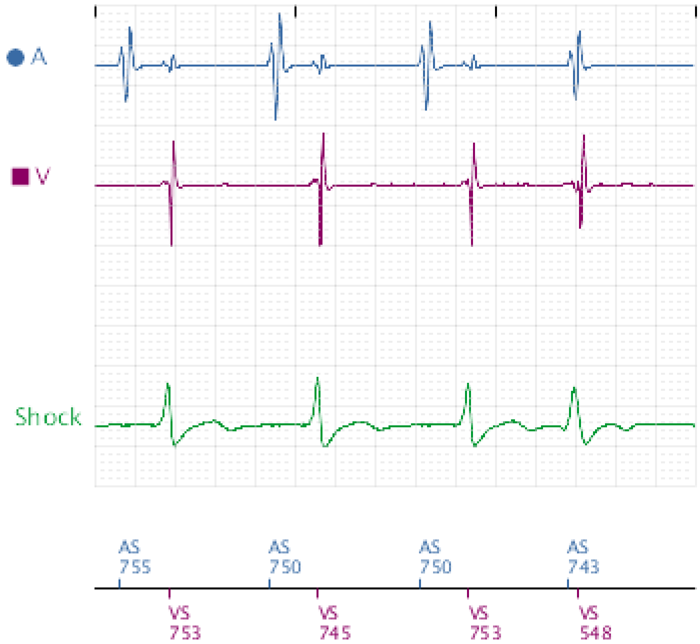

©2013 Boston Scientific Corporation or its affiliates. All rights reserved.

Arrhythmia Logbook Report

LATITUDE Consult™ System - Arrhythmia Logbook Report | **Report Created: Aug [redacted], 2014**

Boston Scientific

[redacted]

Date of Birth: [redacted]
Device: **TELIGEN 100 E110/[redacted]**
Clinic: [redacted] **REGIONAL MEDICAL CE**
Tachy Mode: **Monitor + Therapy**

Latest Device Transmission: **Aug [redacted], 2014 01:17 EDT**
Last Office Interrogation: **May [redacted], 2014**
Implant Date: [redacted] **2012**

Events Since: Implant

Maximum of 250 episodes shown

Event	Date/Time	Type	Therapy	Duration hh:mm:ss
V-6	Jul 31, 2014 20:22	VF	⚠ ATPx1, 41J	00:00:45
V-5	Jul 30, 2014 18:05	VF	ATPx1	00:00:18
V-4	Dec 20, 2013 12:44	NonSustV	Nonsustained	00:00:09
V-3	Dec 03, 2013 17:52	NonSustV	Nonsustained	00:00:07
V-2	Oct 21, 2012 11:15	VF	⚠ ATPx1, 41J, 41J	00:01:00
V-1	Oct 20, 2012 18:53	NonSustV	Nonsustained	00:00:10

©2013 Boston Scientific Corporation or its affiliates. All rights reserved.

Clinician Signature

Event Detail / Episodes Report

(first)

Boston Scientific

[redacted]

Date of Birth: [redacted]
Device: **TELIGEN 100 E110/[redacted]**
Clinic: [redacted] **REGIONAL MEDICAL CE**
Tachy Mode: **Monitor + Therapy**

Latest Device Transmission: **Aug [redacted], 2014 01:17 EDT**
Last Office Interrogation: **May [redacted], 2014**
Implant Date: [redacted] **2012**

V-5: Jul 30, 2014 18:05, VF, A Rate: 86 bpm, V Rate: 224 bpm

Detail

VF Event Onset

Avg A Rate	86 bpm
Avg V Rate	224 bpm
Detection	Rhythm ID

At V-Detect

Avg A Rate	86 bpm
Avg V Rate	220 bpm
Rate Zone	VF
Stability	(3 ms, Off)
V>A Rate	(True, Off)
AFib	(False, Off)
RhythmID Correlated	False
SRD Met	(False, Off)
ATP Timeout	False

Attempt 1, 41 J V Shock

Elapsed Time 00:00:03
Aborted Attempt
VF ATP delivered, no shock attempted due to fail to reconfirm

Event Ended 00:00:18

EGM displayed at 25mm per second

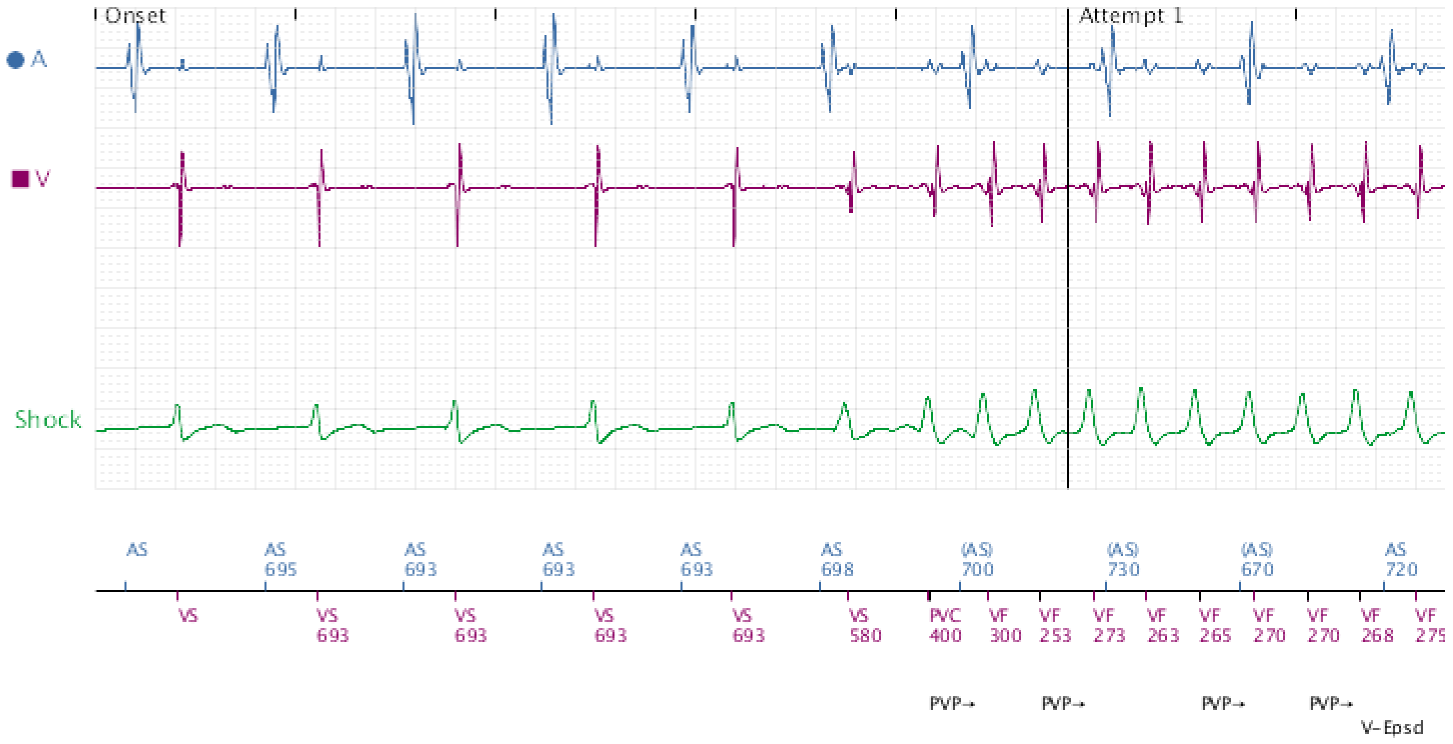

©2013 Boston Scientific Corporation or its affiliates. All rights reserved.

Clinician Signature

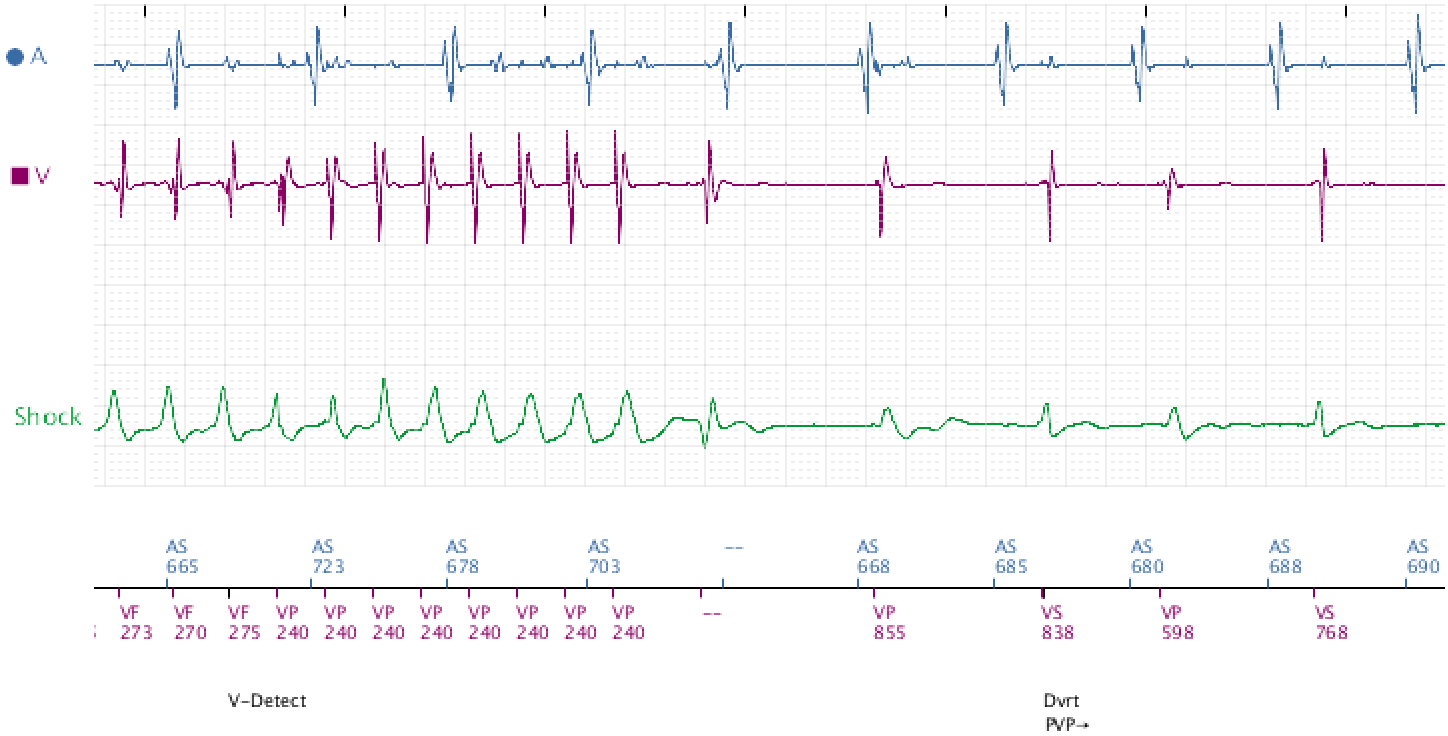

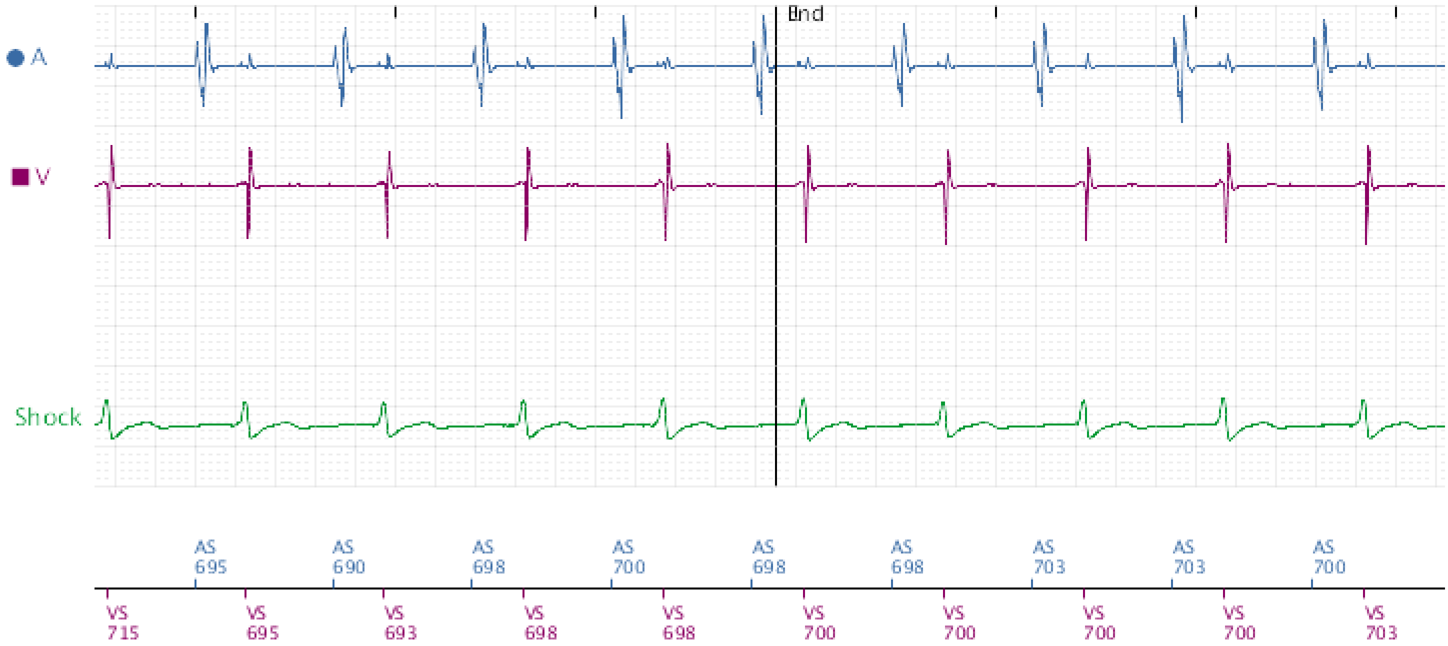

©2013 Boston Scientific Corporation or its affiliates. All rights reserved.

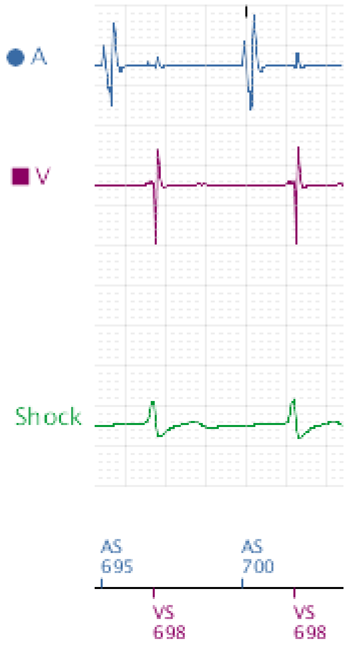

©2013 Boston Scientific Corporation or its affiliates. All rights reserved.

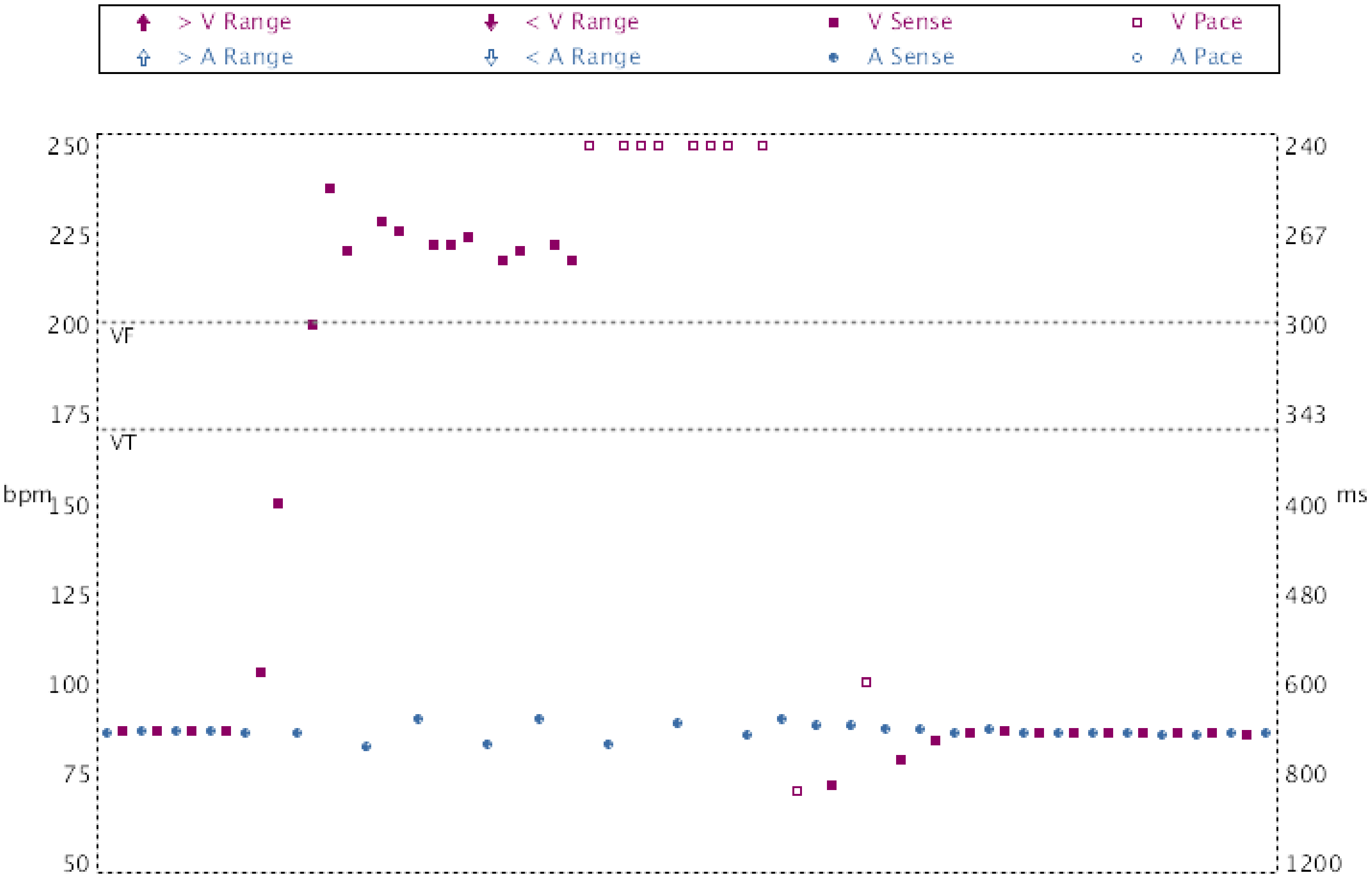

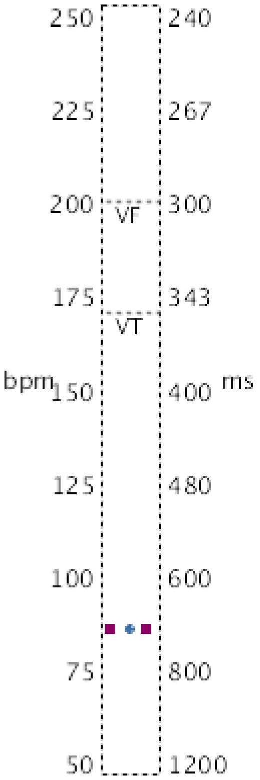

©2013 Boston Scientific Corporation or its affiliates. All rights reserved.

Event Detail / Episodes Report

(second)

Boston Scientific

[redacted]

Date of Birth: [redacted]
Device: **TELIGEN 100 E110/[redacted]**
Clinic: [redacted] **REGIONAL MEDICAL CE**
Tachy Mode: **Monitor + Therapy**

Latest Device Transmission: **Aug [redacted], 2014 01:17 EDT**
Last Office Interrogation: **May [redacted], 2014**
Implant Date: [redacted] **2012**

V-6: Jul 31, 2014 20:22, VF, A Rate: 94 bpm, V Rate: 233 bpm

Detail

VF Event Onset

Avg A Rate	94 bpm
Avg V Rate	233 bpm
Detection	Rhythm ID

At V-Detect

Avg A Rate	94 bpm
Avg V Rate	229 bpm
Rate Zone	VF
Stability	(1 ms, Off)
V>A Rate	(True, Off)
AFib	(False, Off)
RhythmID Correlated	False
SRD Met	(False, Off)
ATP Timeout	False

Attempt 1, 41 J V Shock

Elapsed Time	00:00:03
VF ATP delivered prior to shock	
Shock Information	
Charge Time	8.9 s
Lead Impedance	68 Ω
Lead Polarity	Initial

Event Ended 00:00:45

EGM displayed at 25mm per second

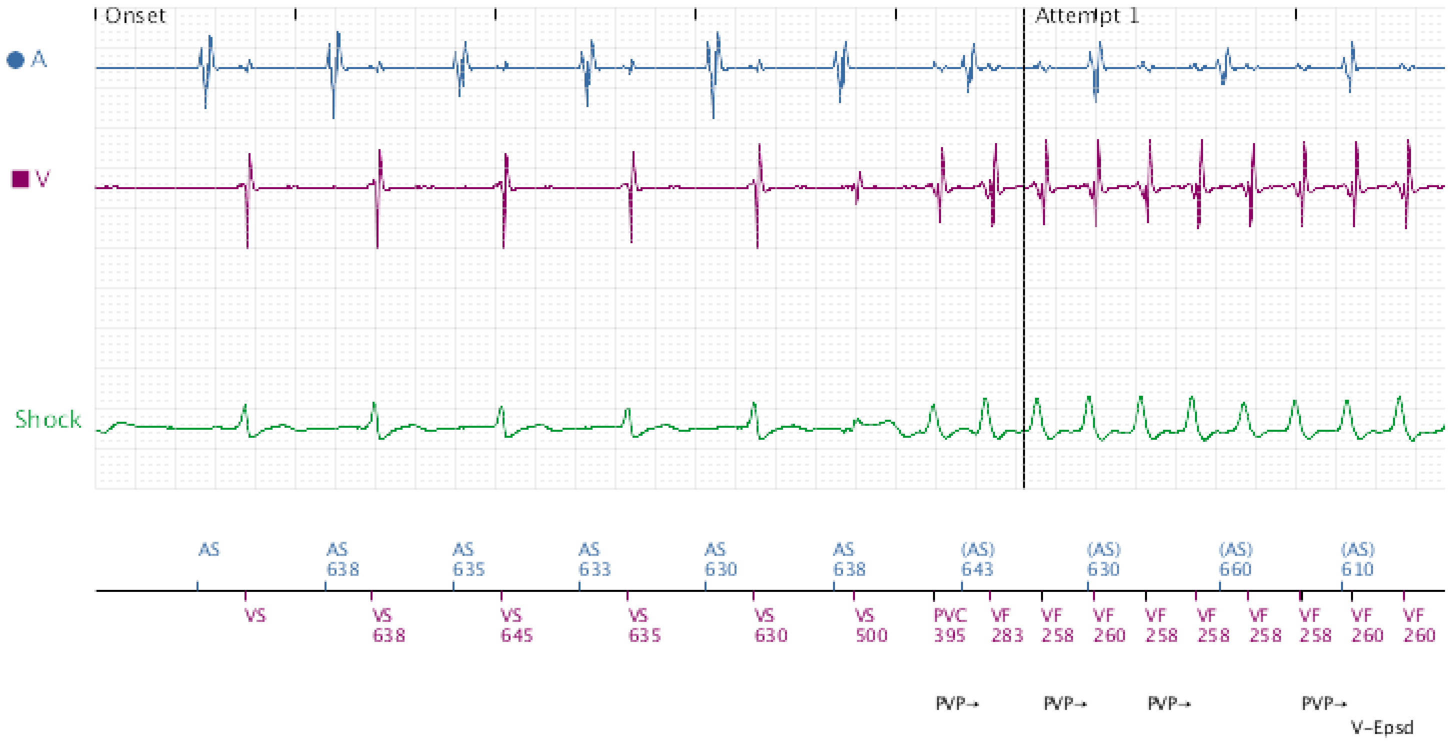

©2013 Boston Scientific Corporation or its affiliates. All rights reserved.

Clinician Signature

[redacted]

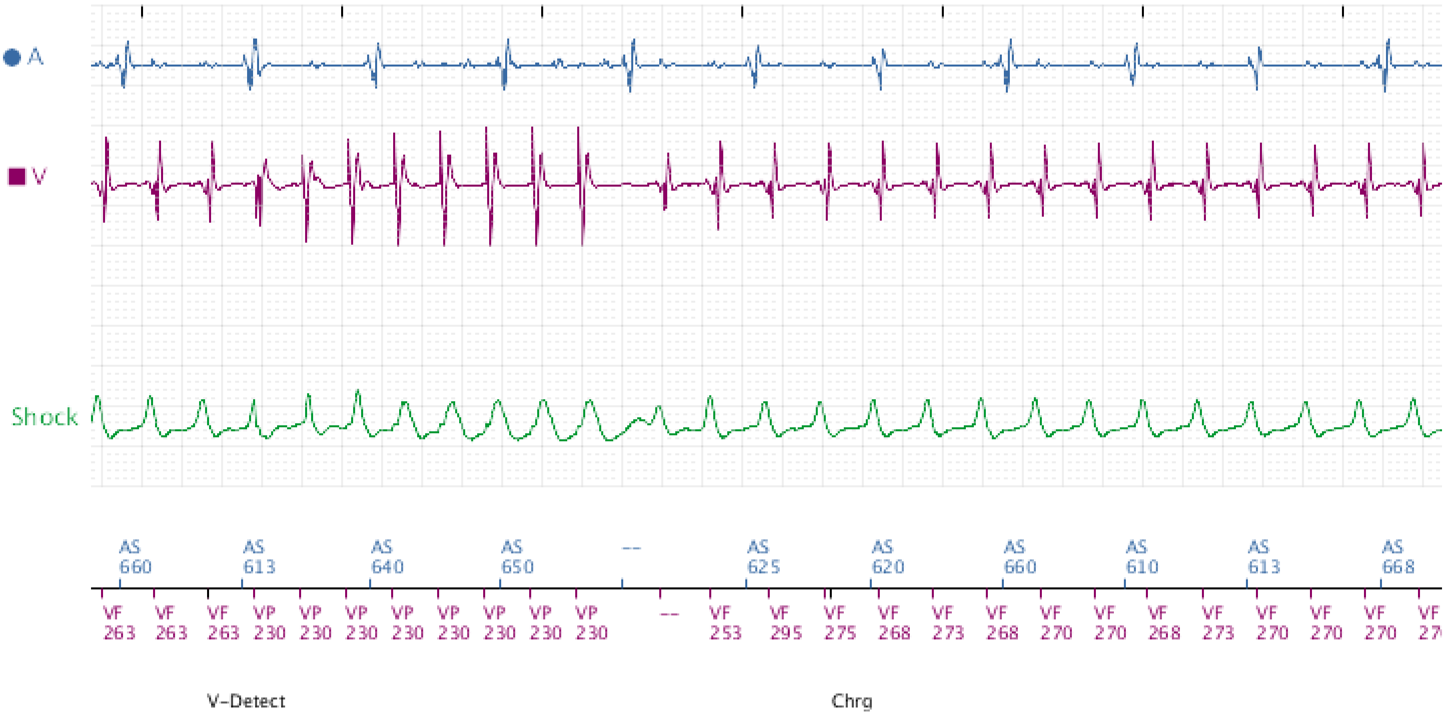

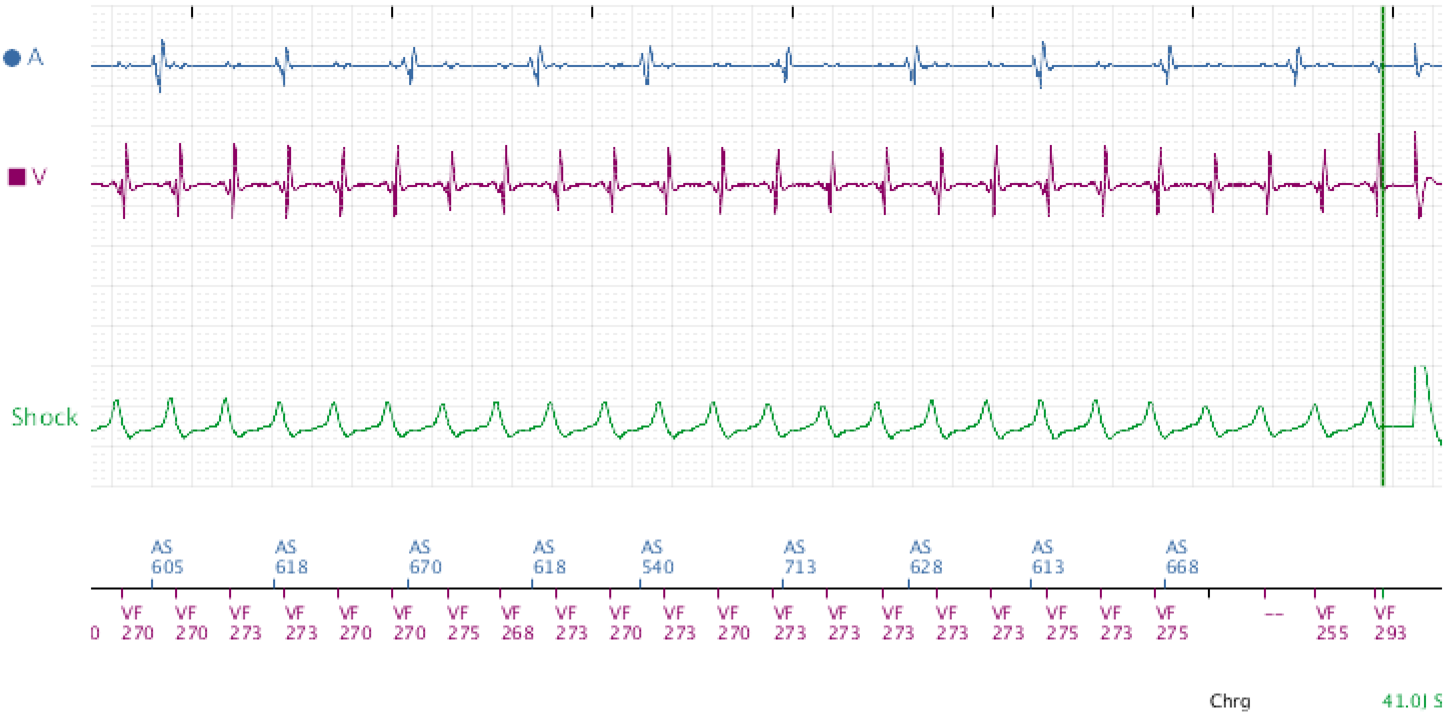

©2013 Boston Scientific Corporation or its affiliates. All rights reserved.

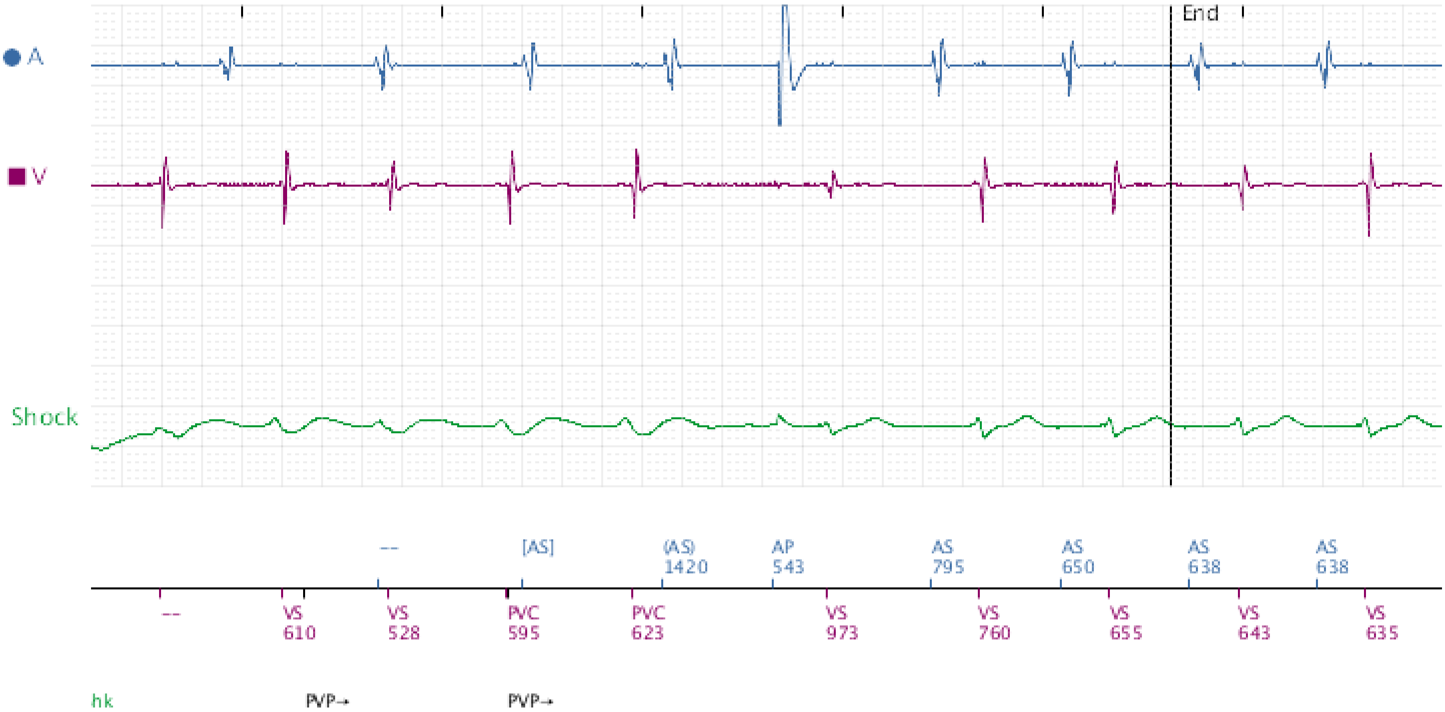

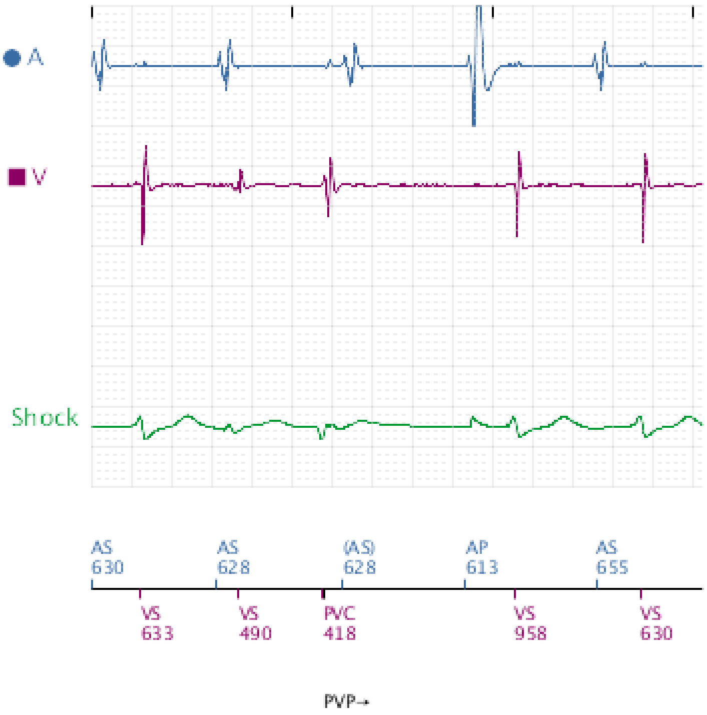

©2013 Boston Scientific Corporation or its affiliates. All rights reserved.

> V Range	< V Range	V Sense	V Pace
> A Range	< A Range	A Sense	A Pace

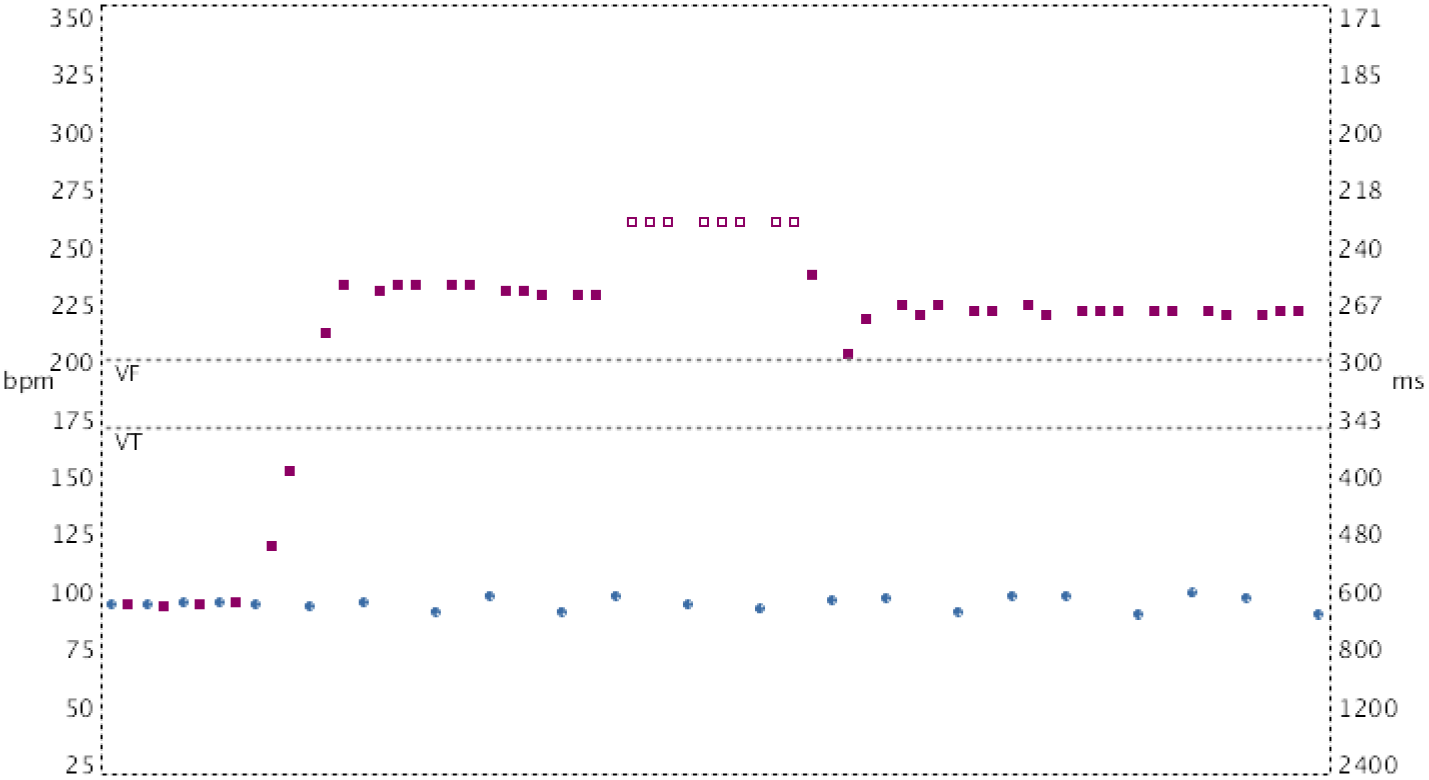

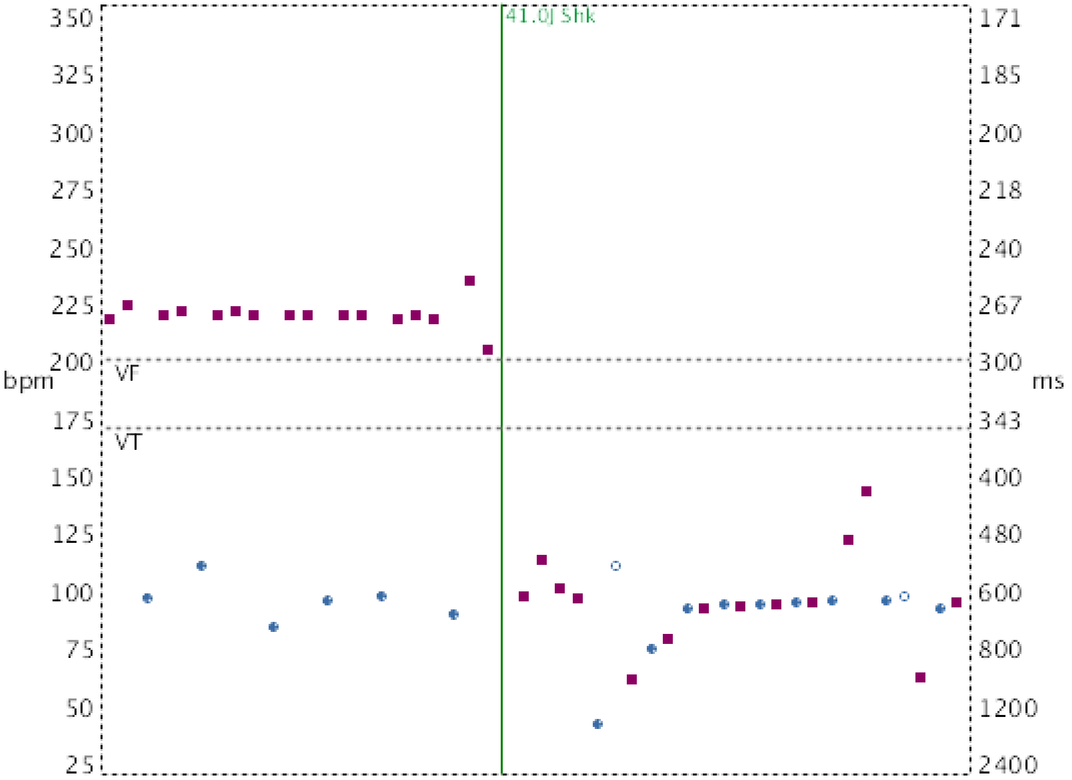

©2013 Boston Scientific Corporation or its affiliates. All rights reserved.

Device Settings Report

[redacted]

Date of Birth: [redacted]
Device: **TELIGEN 100 E110/[redacted]**
Clinic: [redacted] **REGIONAL MEDICAL CE**
Tachy Mode: **Monitor + Therapy**

Latest Device Transmission: **Aug [redacted], 2014 01:17 EDT**
Last Office Interrogation: **May [redacted], 2014**
Implant Date: [redacted] **2012**

Programming

Last Programmed	Feb [redacted], 2013
Ventricular Tachy Mode	[redacted] 2012 Changed to Monitor + Therapy
	[redacted] 2012 Changed to Off

Ventricular Tachy

VF 200 bpm (300 ms)

Detection/Redetection

Initial Duration	1.0 s
Redetection Duration	1.0 s
Post-Shock Duration	1.0 s

Therapy

QUICK CONVERT™ ATP	On
Shock 1	41 J
Shock 2	41 J
Additional 41 J Shocks	6

VT 170 bpm (353 ms)

Detection/Redetection

Initial Duration	2.5 s
Redetection Duration	1.0 s
Post-Shock Duration	1.0 s
**Enhancements**	**Rhythm ID**
**VT Detection**	**On**
**Initial Detection**	**On**
Sustained Rate Duration	03:00 mm:ss
**Post-Shock Detection**	**Off**
**Rhythm ID Setup**	
Passive Method	On
Active Method	On
Temporary LRL	40 ppm
**Common Parameters**	
Atrial Tachy Discrimination	On
AFib Rate Threshold	170 bpm
Stability	20 ms

**ATP1**	**Scan**
Number of Bursts	3
Pulses per Burst	
Initial	8
Increment	0
Coupling Interval	84 %
Decrement	0 ms
Burst Cycle Length	84 %
Ramp Decrement	0 ms
Scan Decrement	10 ms
Minimum Interval	220 ms
**ATP2**	**Off**
**ATP Time-out**	01:00 mm:ss
**Shocks**	
Shock 1	41 J
Shock 2	41 J
Shock 3-6	41 J

Ventricular Tachy Therapy Setup

ATP	
Ventricular ATP Amplitude	7.5 V
Ventricular ATP Pulse Width	1.0 ms
Magnet and Beeper	
Magnet Response	Inhibit Therapy
Beep During Capacitor Charge	Off

Shock (All Shocks)	
Waveform	Biphasic
Committed Shock	Off
Lead Polarity	Initial
Shock Lead Vector	RV Coil to Can

Atrial Tachy

Therapy

ATR Mode Switch Details

ATR Mode Switch	On
Trigger Rate	170 bpm
Duration	0 cycles
Entry Count	8 cycles
Exit Count	8 cycles
Fallback	
Mode	DDIR
Time	00:30 mm:ss
ATR/VTR Fallback LRL	70 ppm
Ventricular Rate Regulation	On
Maximum Pacing Rate	130 ppm

Atrial Tachy Response

Atrial Flutter Response	Off
PMT Termination	On

©2013 Boston Scientific Corporation or its affiliates. All rights reserved.

Clinician Signature

Brady

Normal Settings

Mode	DDD
Lower Rate Limit	40 ppm
Maximum Tracking Rate	130 ppm
Maximum Sensor Rate	130 ppm
Paced AV Delay	280 - 300 ms
Sensed AV Delay	280 - 300 ms
A-Refractory (PVARP)	180 - 280 ms
V-Refractory (VRP)	230 - 250 ms
PVARP after PVC	400 ms
AV Search +	Off
**Blanking**	
A-Blank after V-Pace	Smart
A-Blank after V-Sense	Smart
V-Blank after A-Pace	65 ms
Noise Response	DOO
**Rate Enhancements**	
Rate Smoothing	
Up	Off
Down	Off
Rate Hysteresis	
Hysteresis Offset	Off

Pacing Output

Atrial	3.0 V @ 0.5 ms
Ventricular	2.0 V @ 0.5 ms

Sensitivity

Atrial	AGC 0.25 mV
Ventricular	AGC 0.6 mV

Leads Configuration (Pace/Sense)

**Atrial**	
Pace	Bipolar
Sense	Bipolar
**Ventricular**	
Pace	Bipolar
Sense	Bipolar

Sensor

**Accelerometer**	**ATR Only**
Response Factor	8
Activity Threshold	Medium
Reaction Time	30 s
Recovery Time	2 min
Respiratory Sensor	Off

Post-Therapy Settings

Lower Rate Limit	60 ppm
**Output**	
Atrial	5.0 V @ 1.0 ms
Ventricular	7.5 V @ 1.0 ms
Post Therapy Period	00:30 mm:ss

Note: Post Therapy pacing uses the Brady Normal settings in combination with settings shown in this section.

Setup

Battery Setup

Beeper

Beep when Explant is Indicated	On

Trending Setup

Recording Method	30 Second Average
Duration	25 hours
Data Storage	Continuous

Leads Status Setup

	Daily Intrinsic Amplitude	Daily Impedance	Impedance Limits	Beep When Out-of-Range
Atrial	On	On	200 - 2000 Ω	Off
Ventricular	On	On	200 - 2000 Ω	Off
Shock		On	20 - 125 Ω	Off

©2013 Boston Scientific Corporation or its affiliates. All rights reserved.
